# Uncovering the effects of heterogeneity and parameter sensitivity on within-host dynamics of disease: malaria as a case study

**DOI:** 10.1186/s12859-021-04289-z

**Published:** 2021-07-24

**Authors:** Shade Horn, Jacky L. Snoep, David D. van Niekerk

**Affiliations:** 1 Department of Biochemistry, Stellenbosch University, Private Bag X1, Matieland, 7602 Stellenbosch, South Africa; 2 Molecular Cell Physiology, Vrije Universiteit, De Boelelaan 1087, 1081 HV Amsterdam, The Netherlands

**Keywords:** Within-host, Sensitivity analysis, Malaria, Modelling

## Abstract

**Background:**

The fidelity and reliability of disease model predictions depend on accurate and precise descriptions of processes and determination of parameters. Various models exist to describe within-host dynamics during malaria infection but there is a shortage of clinical data that can be used to quantitatively validate them and establish confidence in their predictions. In addition, model parameters often contain a degree of uncertainty and show variations between individuals, potentially undermining the reliability of model predictions. In this study models were reproduced and analysed by means of robustness, uncertainty, local sensitivity and local sensitivity robustness analysis to establish confidence in their predictions.

**Results:**

Components of the immune system are responsible for the most uncertainty in model outputs, while disease associated variables showed the greatest sensitivity for these components. All models showed a comparable degree of robustness but displayed different ranges in their predictions. In these different ranges, sensitivities were well-preserved in three of the four models.

**Conclusion:**

Analyses of the effects of parameter variations in models can provide a comparative tool for the evaluation of model predictions. In addition, it can assist in uncovering model weak points and, in the case of disease models, be used to identify possible points for therapeutic intervention.

**Supplementary Information:**

The online version contains supplementary material available at 10.1186/s12859-021-04289-z.

## Background

Malaria is a well-known parasitic disease caused by *Plasmodium* parasites and affects populations in tropical and subtropical areas with a significant impact in the sub-Saharan African region. Although an approximate of 228 million cases of infection and a mortality rate of 405,000 has been reported for 2018, the World Health Organization states that the overall incidence of malaria has decreased from 2010 [1]. However, this rate of decrease plateaued with the development of resistance to current treatments, necessitating new investigative approaches into disease treatment and eradication [1, 2]. A well-studied part of the parasite life-cycle is the blood stage within the human host [3]. In this erythrocytic stage, merozoites infect red blood cells, where they mature and proliferate until the red blood cells burst and release more merozoites [4]. The erythrocytic stage is associated with clinical symptoms of malaria since parasites and infected red blood cells (iRBCs) activate the immune system’s response, which can lead to symptoms such as fever, malaise and exacerbate anaemia [5]. The immune system, however, plays an important role in disease progression and response to treatment and vaccination. Taking its response into consideration is therefore vital for a quantitative understanding of infection dynamics and intervention effects [5].

Upon malaria infection, the always-present innate immune system rapidly attempts elimination of the invading pathogen [6, 7]. The innate immune system is non-specific to an infection and fights microbes by secreting various proteins and cytokines and assisting the adaptive immune system [8]. The adaptive immune response takes longer to develop and can be sub-divided into two categories, namely humoral and cell-mediated immune responses [7–9]. Here the humoral response would target free pathogens in the blood using antibodies produced from B-lymphocytes, whereas the cell-mediated response targets infected cells with T-cells differentiated from T-lymphocytes. Even with these multiple immune responses, malaria infection can persist in the body leading to severe complications and death [10].

Experimental methods such as bioluminescent, behavioural studies and a variety of assays can be used to investigate the malaria parasites, their interactions with the human and mosquito hosts and the immune system’s response to infection [6, 11, 12]. Clinical studies are also used to investigate parasite interactions and treatment effects, using methods such as microscopy, rapid diagnostic tests and molecular assays [13]. Data from clinical and experimental observations can be encoded in mathematical models. These models can then be used to analyse the behaviour emerging as a function of interacting processes in the system, and to make quantitative predictions of how a system is influenced by various alterations. A variety of within-host mathematical models describing the disease dynamics associated with malaria infection exist, with differential equation based models focusing on the time evolution of different cell populations within the host [14–37]. Simple models of the erythrocytic infection stage usually describe populations of healthy red blood cells (RBCs), infected red blood cells (iRBCs) and free roaming merozoites with some of these models extended to include immune system components [21–37].

When comparing these models it becomes evident that they often differ in their formalism, structure and in the interpretation of model components and simulation results depending on the purpose of the original study. In each model, the parametrisation of the biological processes approximate the dynamics that might only be realistic close to the reference state. In addition, the values of parameters could be imprecise due to the method of determination employed and a natural variation in values can occur in a population. Consequently, there is some uncertainty regarding the reliability and fidelity of the model predictions and their interpretations. For the purpose of this study we interpret reliability as the extent to which model predictions can be trusted in the context of parameter uncertainty, and fidelity as the degree to which model predictions reflect reality taking heterogeneity in a population into account.

Various methods exist to quantify the effects of parameter variations or changes within a model. These can be used to determine the contribution of parameter uncertainty to variance in model prediction, to test model robustness against parameter changes and even assist in elucidating biologically relevant constituents for intervention with a certain outcome in mind.

In a biological system such as the human body, there is an expected range in which population sizes of cells may fall, as a natural variance will be seen in a population of individuals with slightly different characteristics (i.e. parameter sets). Robustness analysis can demonstrate the possible model outcomes for individuals in a population where biological parameters can vary greatly between individuals. A robust model shows resilience to changes in model inputs, presenting a more stable model [38, 39], although it should be able to account for variances seen in a population. If clinical ranges of observables (such as cell types) are available this analysis can also establish a degree of confidence in the fidelity of model predictions given the variation seen in a population of heterogeneous individuals.

Considering the experimental uncertainty in parameter values, uncertainty analysis allows one to quantify the contribution of uncertainty of a parameter to the overall uncertainty in model predictions [40]. Parameters are often obtained from literature where clinical measurements were pooled in studies not designed for model parametrisation. Uncertainty analysis can thus indicate which parameters have a large effect on model outputs when considering their variances.

Local sensitivity analysis is closely associated to uncertainty analysis, as it entails determining the change in model outputs (e.g. steady state values of model variables) when the inputs or parameters are varied one at a time in a localised parameter space around the reference state [41]. The method is applied to each parameter individually, while the rest are kept at the wild type values, and the results, shown as sensitivity indices, quantify the effect of each parameter on model outcomes near the reference state. Whereas uncertainty analysis indicates which parameters lead to the most uncertainty in model outputs given their variances, local sensitivity analysis quantifies the sensitivities of model outputs to small parameter perturbations around a specific point in parameter space. For a review of possible applications of this analysis see [42, 43]. In the context of metabolic systems, metabolic control analysis (MCA) is a form of local sensitivity analysis which entails calculating normalised partial derivatives of the model outputs (e.g. steady state concentrations or fluxes) with respect to system properties (rates, concentrations or parameters) [44–47]. In MCA the response coefficient is a sensitivity index quantifying the fractional change in the steady state outputs of the model variables or fluxes upon a 1% change in a parameter [47]. Beyond model sensitivity characterisation, this approach can also be used to indicate possible drug targets and their systemic effects [48–50] if one considers parameters with large responses to be potential weaknesses in the system.

The last type of analysis of interest here is a variation on robustness analysis where local sensitivity coefficients are calculated at each point in the complete parameter space. Ideally the parameter ranges should be defined according to the ranges observed in a population. Random parameter sets are constructed and the model simulated for each set - similar to the robustness analysis described above. The local sensitivities (response coefficients) are determined for every parameter set (point in parameter space) and the results are pooled to visualise the spread of response coefficients (sensitivities) in a population. For this analysis the results can be visualised using histograms, and good robustness of local sensitivities in a population is inferred when the results are well conserved with the most probable response coefficient corresponding to the wild-type result from local sensitivity analysis. This analysis can hence assist in determining if the results of the wild-type local sensitivity analysis are approximately retained in a heterogeneous population [48]. As this analysis samples the parameter set from the global parameter space, it could be considered a form of global sensitivity analysis, but since the aim is to analyse the robustness of the local sensitivity results, it will be defined as local sensitivity robustness analysis in this study. Global sensitivity analysis methods used on biological models as described in [47] have different levels of computational and mathematical complexity. It would however be best to use methods that closely relate to the methods used for robustness and local sensitivity analyses for comparison.

This study focusses on analysing four published models of malaria infection where the immune system’s response is incorporated. The models were chosen based on their ability to describe the core dynamics of the disease with varying complexity of the immune system description. Additional considerations were their ordinary differential equations (ODE) structure, and comparability of model variables and processes. Uncertainty, robustness and sensitivity analyses were performed on these within-host models to determine the effect of parameter uncertainty and variability on the predicted disease dynamics, and to test whether the models could still give reliable and realistic predictions while accommodating heterogeneity and uncertainty.

## Model descriptions

The model of Anderson et al. [21] is one of the earliest models on which many others have built. This model includes four variables, describing the RBC, iRBC, merozoite and T-lymphocyte populations, where the T-lymphocyte population represents the immune effectors of the model. The model of Li et al. [22] has a very similar, albeit expanded model structure to that of Anderson et al. [21]. It includes immune effector parameters in Michaelis–Menten–Monod functions to ensure saturation of processes corresponding to the immune system’s response. Niger and Gumel [23] extended the model of Anderson et al. [21] by partitioning the immune system response into two variables: the collective immune effectors and the antibodies specific to malaria infection. The model furthermore separated the iRBC population into different compartments, to account for parasite growth. The final model included for analysis is from the doctoral thesis of Okrinya [24]. Whilst also splitting the immune effectors into two groups as in the Niger and Gumel [23] model, the two immune response variables in this model denote innate and adaptive immunity. It includes an additional variable representing the gametocyte population within the host. The model structures are explained in the following section, and parameter definitions used to reproduce the models prior to analysis can be viewed in Additional file 1: Tables 1–4. It should also be noted that multiple model outputs describing different disease states were obtained in some publications, where parameter values were altered to showcase either parasite free or endemic states. As we are interested in investigating the parameter effects on disease, only models describing infection were used.

### Anderson model

Anderson et al. [21] formulated a within-host model of blood stage malaria infection, which includes the immune response to free roaming merozoites and iRBCs. The model construct with RBCs (*x*), iRBCs (*y*), merozoites (*s*) and T-lymphocytes (*T*) follows:

1a $$\begin{aligned} \frac{dx}{dt}&=\lambda -\mu {x}-\beta {x}{s} \end{aligned}$$

1b $$\begin{aligned} \frac{dy}{dt}&=\beta {x}{s} - \alpha {y} -{g}{y}{T} \end{aligned}$$

1c $$\begin{aligned} \frac{ds}{dt}&=\alpha {r}{y} -{d}{s} -\beta {x}{s}-{h}{s}{T} \end{aligned}$$

1d $$\begin{aligned} \frac{dT}{dt}&=\gamma {s}{T} + {k}{y}{T} -{a}{T} \end{aligned}$$

The model shows the natural birth rate of healthy RBCs, $\lambda$, and natural death rates $\mu x$, $\alpha y$, *ds* and *aT* for RBCs, iRBCs, merozoites and T-lymphocytes respectively. $\beta {x}{s}$ is a transfer term present in three equations, where $\beta$ denotes the probability of a merozoite infecting a healthy RBC. Thus, this term depends on, and influences merozoite (*s*), as well as available RBC population densities (*x*). The term $\alpha {r}{y}$ describes the increase of merozoites due to the death rate of iRBC, where the cells burst and release *r* number of merozoites in the blood. The immune system component decreases the iRBC and merozoite densities with rate constants *g* and *h* respectively, whilst also being activated by the iRBCs and merozoites with rate constants *k* and $\gamma$. Although this model specifies that T-lymphocytes are the immune effectors used to fight infection, they affect both merozoites and iRBCs. This indicates humoral and cell-mediated immunity with humoral immunity fighting against merozoites and cell-mediated immunity defending against iRBCs. This model, however, does not include an innate immune response as there is no natural birth rate for immunity effectors. Four different sub-models were published based on this model where the last model as shown here uses the complete structure where humoral and cell-mediated immunity is included.

### Li model

Li et al. [22] contains four equations for variables *H* (RBCs), *I* (iRBCs), *M* (merozoites) and *E* (immune effectors). Here “immune effectors” include all biological immune effectors and no distinction is made between innate and adaptive immunity.

2a $$\begin{aligned} \frac{dH}{dt}&=\lambda -{d_1}{H}-\alpha {H}{M} \end{aligned}$$

2b $$\begin{aligned} \frac{dI}{dt}&=\alpha {H}{M} -\delta {I}-\frac{{p_1}{I}{E}}{1+\beta {I}} \end{aligned}$$

2c $$\begin{aligned} \frac{dM}{dt}&=r{I}-\mu {M}-\frac{{p_2}{M}{E}}{1+\gamma {M}} \end{aligned}$$

2d $$\begin{aligned} \frac{dE}{dt}&=-{d_2}{E}+\frac{{k_1}{I}{E}}{1+\beta {I}}+\frac{{k2}{M}{E}}{1+\gamma {M}} \end{aligned}$$

This model extends the Anderson model, by incorporating non-linear bounded Michaelis-Menten-Monod functions to account for saturation of the immune-linked elimination processes. The first process of this kind is the removal of the iRBCs (*I*) by immune effectors *E*, represented by $\frac{{p_1}{I}{E}}{1+\beta {I}}$ in Eq. 2b. In this term $p_1$ is a rate constant for the rate at which immune effectors can remove iRBCs, and $\frac{1}{\beta }$ is viewed as a half-saturation constant for iRBCs. The same holds true for the term $\frac{{p_2}{M}{E}}{1+\gamma {M}}$, where $p_2$ is the rate at which the immune effectors can remove the merozoites in the blood plasma, while $\frac{1}{\gamma }$ is a half-saturation constant. $k_1$ and $k_2$ describe the proliferation rate of lymphocytes due to activation by iRBCs and merozoites, respectively. The immune response is split into two components in the relevant equation 2d. Here the second term corresponds to the immune response component that proliferates due to the activation by iRBCs. The third term corresponds to the immune response component that is activated by merozoites. The merozoites are eliminated by the humoral immune effectors (rate constant $p_2$) and the iRBCs by the cell-mediated immune effectors (rate constant $p_1$). Immune effector activation is a saturable process and depends on the population density of the disease variables as well as the immune cell concentration, their binding and the efficiency of the process. Six sub-models were published with the same structure but changes were made in key parameters to obtain different disease dynamics. The one chosen here for analysis describes an endemic state of malaria infection where the immune system’s response is included.

### Niger model

Niger et al. [23] contains age compartments ($Y_i$) of the intracellular parasite stage (iRBCs), representing the different stages of parasite growth in an infected erythrocyte. The authors also proposed a more physiologically realistic model which splits the immune effectors into two groups: immune cells *B* and antibodies *A*. The model, which includes healthy RBCs (*X*) and merozoites (*M*), follows:

3a $$\begin{aligned} \frac{dX}{dt}&=\lambda _X - \beta {X}{M}-\mu _X{X} \end{aligned}$$

3b $$\begin{aligned} \frac{dY_1}{dt}&=\beta {X}{M}-\mu _1{Y_1}-\gamma _1{Y_1}-k_1{B}{Y_1} \end{aligned}$$

3c $$\begin{aligned} \frac{dY_2}{dt}&=\gamma _1{Y_1}-\mu _2{Y_2}-\gamma _2{Y_2}-k_2{B}{Y_2} \end{aligned}$$

3d $$\begin{aligned} \vdots \\ \frac{dY_n}{dt}&=\gamma _{n-1}{Y_{n-1}}-\mu _n{Y_n}-\gamma _n{Y_n}-k_n{B}{Y_n} \end{aligned}$$

3e $$\begin{aligned} \frac{dM}{dt}&=r(\mu _n + \gamma _n){Y_n}- \mu _M{M} - k_M{B}{M} - \mu {\beta }{X}{M} \end{aligned}$$

3f $$\begin{aligned} \frac{dB}{dt}&=\lambda _B + B(\rho _1{Y_1} + \rho _2{Y_2}+ \cdots + \rho _n{Y_n} + \rho _{n+1}{M}) - \mu _B{B} \end{aligned}$$

3g $$\begin{aligned} \frac{dA}{dt}&=\eta {B}{M} - \mu _A{A} \end{aligned}$$

For the first stage of infection $Y_1$, Eq. 3b shows infection of healthy RBCs dependent on $\beta$, the infection rate constant, as well as the variables *X* and *M*, depicting the healthy RBCs and merozoite population, respectively. The natural death rate $\mu _i{Y_i}$ of iRBCs and the progression rate to the next compartment, $\gamma _{i}{Y_{i}}$, both decrease the $Y_i$ population in each compartment. The immune system now additionally kills iRBCs, where $k_i$ is the immuno-sensitivity of the stage *i* iRBCs to immune effectors *B*. In this model *n* covers five stages where the parameter values differ slightly as shown in Appendix Table 3. *B* accounts for immune cells including the innate immune effectors, whereas *A* accounts for merozoites specific antibodies. This distinction is important as innate immune cells are ever-present, whereas antibodies are only formed when the acquired immune response is activated. For the immune cells *B* there is a natural background production rate of cells, $\lambda _B$, as well as a stimulation of production rate due to the presence of an infection (at a rate $\rho _i{Y_i}$). This stimulation of the production happens due to all infected compartments including free merozoites. For antibodies there is only an increase of antibodies at a rate $\eta {B}{M}$, dependent on the immune effector and merozoite populations. However, in this model the antibodies (*A*) are not included in any other processes and is therefore not integrated into the immune response that influence infection. The model used here shows a stable endemic state with an immune response.

### Okrinya model

The Okrinya model [24] has an extra variable *G* for gametocyte population. The model with uninfected RBCs (*X*), iRBCs (*Y*), merozoites (*M*), innate immune cells (*P*) and adaptive immune cells (*A*), is given by:

4a $$\begin{aligned} \frac{dX}{dt}&=\lambda _x -\frac{ \beta _x{X}{M}}{1+c_0{A}}-\mu _x{X} \end{aligned}$$

4b $$\begin{aligned} \frac{dY}{dt}&=\frac{ \beta _x{X}{M}}{1+c_0{A}} - (\mu _y + \mu _n){Y} -k_y{P}{Y}(1+k_a{A}) \end{aligned}$$

4c $$\begin{aligned} \frac{dM}{dt}&=\frac{r{\mu _y}{(1-\theta )}{Y}}{1+c_1{A}} - \frac{\beta _x{X}{M}}{1+c_0{A}} - \mu _m{M} - k_m{P}{M}(1+k_b{A}) \end{aligned}$$

4d $$\begin{aligned} \frac{dG}{dt}&=\frac{r{\mu _y}{\theta }{Y}}{1+c_1{A}} - \mu _g{G} - k_g{P}{G}(1+k_c{A}) \end{aligned}$$

4e $$\begin{aligned} \frac{dP}{dt}&=b_m + \eta _1(Y +\phi {M}) - \mu _p{P} -P(k_d{Y} + k_n{M}) \end{aligned}$$

4f $$\begin{aligned} \frac{dA}{dt}&=\eta _2\{Y(t-d_1) + g_2{M}(t-d_1)\} +\mu _a{(A_0 - A)} - A(\eta _3{Y} + \eta _4{M}) \end{aligned}$$

The effector variable *A* represents the population of adaptive immune cells and include cells like B and T-cells, but the author interprets it as the concentration of antibodies. The immune effectors are thus split differently compared to the Niger model. Comparison to the structure of the Niger model also shows the differences in processes. For example, terms that include $\frac{1}{(1+c_1{A})}$ indicate the effect of antibodies blocking the infection. The term ${k_m}{P}{M}(1+k_b{A})$ depicts the immune system’s successful removal of merozoites, which is dependent on the concentrations of both the innate immune cells and merozoites. An extra parameter $\theta$ in the equations for *M* and *G* (Eq. 4c and 4d) is the fraction of merozoites that will develop into gametocytes and therefore not re-enter the erythrocytic cycle. As was seen for the Niger model, innate immune cells have a natural birth rate and a stimulation rate bought on by infection ($\eta _1$), whereas the adaptive immune effectors are only produced when there is an actual presence of disease. Note that a time delay ($t-d_1$) is included in the equation, to account for the interval between start of infection and production of adaptive immune cells. This time delay affects the dynamics upon infection but does not influence the achieved variable steady state values, and was ommited in our reproduction to simplify analysis. The model, describing pathogenesis in an infected individual with an immune response, was published in a dimensional and non-dimensionalised form. To enable the comparison of this model to the others, the dimensional model was used.

### General remarks on model reproduction

Models were reproduced from literature using the published parameters values as the reference/wild-type set. Simulation results for steady state values of variables and dynamic behaviour were compared to published results and showed good agreement if not exact. It should be noted that for the Li model, some parameter sets used in a Monte Carlo simulation presented here, resulted in oscillatory behaviour (limit cycle). In these cases, our analyses were performed using the unstable steady state.

Parameter symbols and definitions differ between models. Biologically similar parameters were therefore first identified and for the purposes of this study they are denoted by the same symbol (which might differ from the original symbols used in the models) as shown in Table 1. All parameter definitions and values for each model can be viewed in Additional file 1: Tables 1–4.

**Table 1:** Relatable parameters between models

	Anderson	Li	Niger	Okrinya
Elimination rate of merozoites by immune effectors	*h*	$p_2$	$\varvec{k_M}$	$\varvec{k_M}$
Probability of infection of RBCs with free roaming merozoites	$\varvec{\beta }$	$\alpha$	$\varvec{\beta }$	$\beta _X$
Production rate of immune cells	.	.	$\varvec{\lambda _B}$	$b_M$
Birth rate of healthy RBCs	$\lambda$	$\lambda$	$\varvec{\lambda _X}$	$\varvec{\lambda _X}$
Natural death rate of RBCs	$\mu$	$d_1$	$\varvec{\mu _X}$	$\varvec{\mu _X}$
Natural death rate of iRBCs	$\alpha$	$\delta$	$\mu _{Y_5}$	$\varvec{\mu _N}$
Death rate of innate immune cells / immune effectors	*a*	$d_2$	$\mu _B$	$\varvec{\mu _P}$
Natural death rate of merozoites	*d*	$\mu$	$\varvec{\mu _M}$	$\varvec{\mu _M}$
Proliferation rate of immune effectors due to merozoites	$\gamma$	$\varvec{k_2}$	$\rho _6$	$\eta _1\phi ^{\mathrm{a}}$
Proliferation rate of immune effectors due to iRBCs	*k*	$\varvec{k_1}$	$\rho _1$	$\eta _1$

A parameter definition is given in terms of the rate in which it appears with the corresponding parameter symbol from each model. The symbol used to represent this parameter in the results section is emphasized in bold

$^{\mathrm{a}}$treated as a single parameter for the analyses conducted in the current study

## Results

### Robustness analysis

**Fig. 1:**
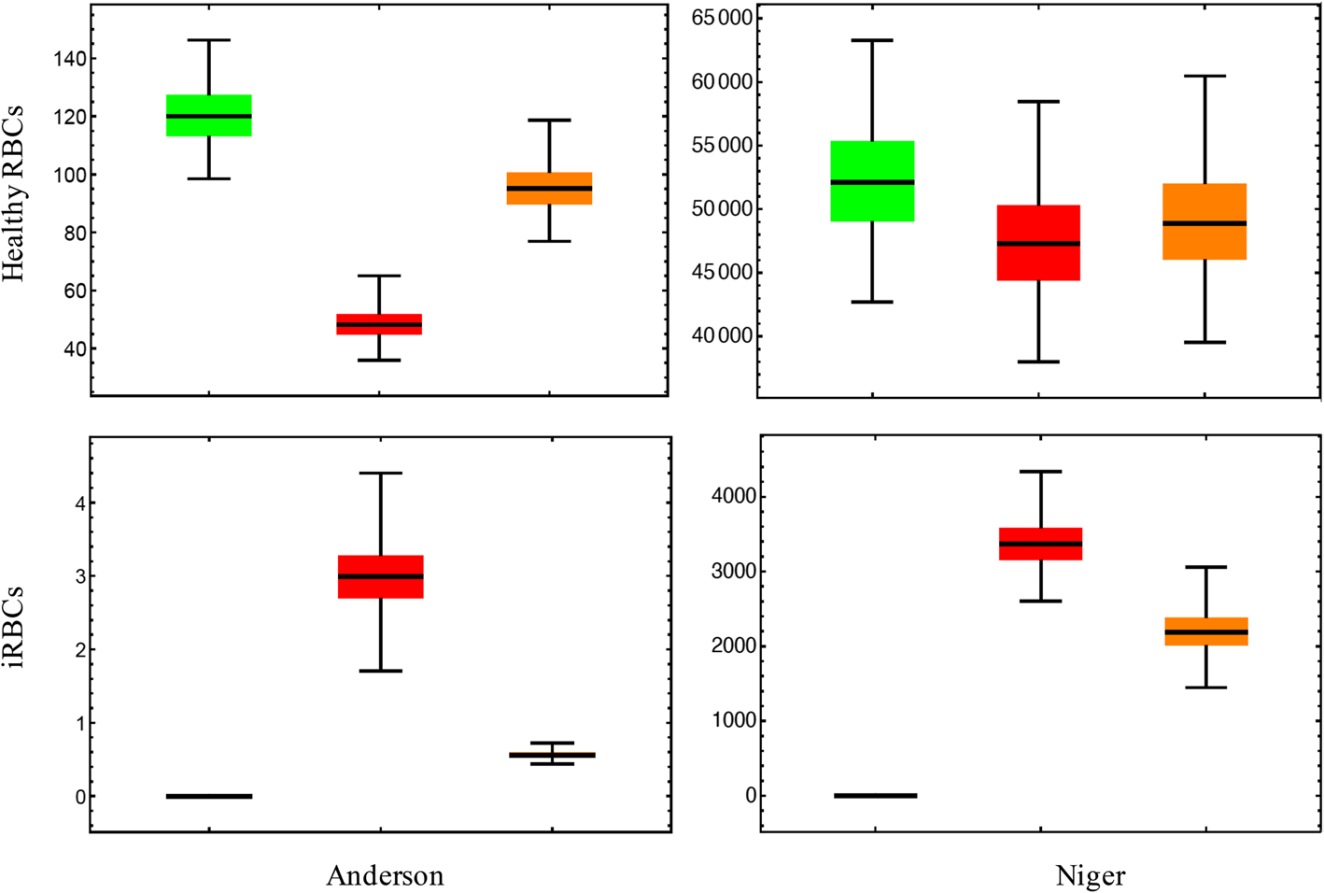
Robustness analysis results for the infected and healthy RBC populations of the Anderson and Niger models. Three different disease states are shown in the diagrams: (i) green—no infection, (ii) red—malaria infection without an immune response and, (iii) orange—infection with an immune response. The steady state values for the healthy RBCs decrease with infection in both models and increase again with the addition of the immune system. The values for the iRBCs start as none with no infection, increase to the highest achieved results when infection is added, and decrease again when the immune system is included in the model

Figure 1 shows the range of endemic steady state values for RBCs and iRBCs for 10,000 parameter sets obtained by a Monte Carlo (MC) random sampling-based technique. All parameters were randomly varied within $10\%$ of their wild-type values and the steady state values determined for each parameter set. The results for the models of Anderson and Niger show three different disease states i.e. a healthy patient (green), an infected patient with no immune response (red) and an infected patient with an immune response (orange).

Note that the healthy RBCs and iRBCs are unitless in Fig. 1, as Anderson published units only in 1/*day* and Niger published units in “concentration”. With no infection (green) the healthy RBCs achieve the highest steady state values. These values are shown to drastically decrease with infection (red) and then increase with the addition of the immune response (orange). Although the values of the steady states reached for the infected population with the inclusion of the immune system is higher than those for infection without an immune response, the uninfected steady state values are not recovered. For the iRBCs there are no steady state values in an uninfected individual, non-zero steady state values when there is infection, and subsequently decreased steady state values with the added immune response. These results were shown to indicate the differences between disease states but as is evident, the values reached at steady state for the healthy and infected RBCs in the Anderson model are extremely low. These plots are therefore only to be used as a general representation of how these variable distributions will shift upon infection and the inclusion of the immune response. The general trend of the healthy and infected RBCs as seen for Fig. 1, where, for example, the healthy RBCs decrease with infection and then increase with the inclusion of the immune response, was present in all models with the simulation of the different disease states.

Table 2 shows the steady state value ranges obtained for all variables in each of the models with infection and an immune response. Note that immune effectors include cells from the innate and adaptive immune system. Antibodies (*A*) from the Niger model is not included in the immune effectors shown here as they are not classified as cells and the immune effectors for the Okrinya model includes only the innate immune cells. Robustness analysis should give all models 10,000 parameter sets with an equivalent number of steady state values per variable, however, some of the Li parameter sets resulted in unphysical steady state values (negative concentrations), possibly due to the instability mentioned in the “General remarks on model reproduction” section above, and were excluded from the results. To compare model outputs using the same number of parameter sets, the Li model therefore required additional sampling to obtain a total of 10,000 valid parameter sets.

**Table 2:** The ranges of the steady state values for all variables obtained during robustness analysis

	Anderson$^{\mathrm{a}}$	Li	Niger$^{\mathrm{a}}$	Okrinya
RBCs	$7.59\times 10^1-$	$3.73 \times 10^6 -$	$3.97 \times 10^4 -$	$1.12 \times 10^6 -$
	$1.18 \times 10^2$	$5.52 \times 10^6$	$6.00 \times 10^4$	$2.27 \times 10^6$
iRBCs	$4.26\times 10^{-1}-$	$2.40 \times 10^3 -$	$1.47 \times 10^3 -$	$2.73 \times 10^4 -$
	$7.14 \times 10^{-1}$	$8.77 \times 10^3$	$3.07 \times 10^3$	$5.37 \times 10^4$
Merozoites	$1.62\times 10^{-2}-$	$5.73 \times 10^2 -$	$3.77 \times 10^1 -$	$2.11 \times 10^3 -$
	$2.91 \times 10^{-2}$	$2.14 \times 10^3$	$1.07 \times 10^2$	$5.87 \times 10^3$
Immune cells	$1.27\times 10^{0}-$	$6.86 \times 10^4 -$	$4.44 \times 10^1 -$	$1.21 \times 10^{-1} -$
	$6.87 \times 10^{0}$	$1.60 \times 10^8$	$7.11 \times 10^1$	$1.78 \times 10^{-1}$ $^{\mathrm{b}}$
Parasitemia ($\%$)	$0.571 -$	$0.066 -$	$3.42 -$	$2.31 -$
	0.614	0.154	4.86	2.51

Variables have units of ${\mathrm{{cells}}}/\upmu {\mathrm{{l}}}$

$^{\mathrm{a}}$The Anderson and Niger models were published without units

$^{\mathrm{b}}$The adaptive immune cells in the Okrinya model (defined as “antibodies” is in ${\mathrm{{mol}}}/{\mathrm{{cell}}}$, whereas the innate immune cells are in ${\mathrm{{cells}}}/\upmu {\mathrm{{l}}}$. As such only the innate immune cell range is shown in the table for unit comparability. The range of the antibodies is $2.73 \times 10^{-2} - 6.42 \times 10^{-2} \;{\mathrm{{mol}}}/{\mathrm{{cell}}}$

Of all the models the Li model showed the best correlation to known clinical ranges for the healthy RBC population in infected individuals (4.41–6.48$\times 10^6 \;{\mathrm{{cells}}}/\upmu {\mathrm{{L}}}$) [51]. Immune effectors in the models do not corresponded to the published range of white blood cells (4.52–7.99$\times 10^3 \;{\mathrm{{cells}}}/\upmu {\mathrm{{L}}}$) [52]. It is also noteworthy that the range for the Li model’s immune effectors is very large, showing that the immune effectors are very sensitive to changes in the model parameters, which could also affect all other variables. The $\%$ parasitemia ranges can be calculated from these results using $100\cdot \frac{iRBCs}{iRBCs+RBCs}$, for simulation results from the same parameter set. Given that severe malaria is classified as $>5\%$ parasitized cells [53, 54], all models indicate low to moderate parasitemia with the Niger model verging the closest to severe malaria.

**Fig. 2:**
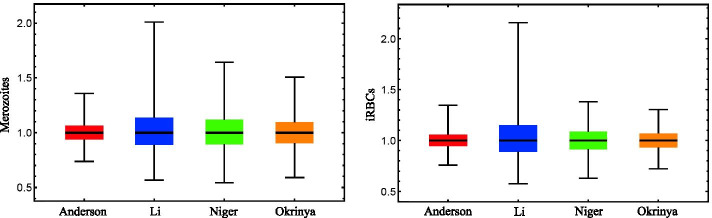
Robustness analysis results for the merozoites and iRBCs of all four models. The results were normalized to the median and shows the distributions of the disease variables. Robustness decreases in the order: Anderson (red) > Okrinya (orange) > Niger (green) > Li (blue)

For the robustness results in Fig. 2, each model’s results were normalized to their median as all four models achieved different ranges of steady state values.

Although the parameter sets are obtained from uniform distributions, robustness analysis results show more of a normal distribution, indicating good robustness for all models. The Anderson model appears to be the most robust for the merozoites, with the Li model showing a wider range for both the merozoites and iRBCs. The Okrinya and Niger models also show good robustness for the iRBCs and merozoites.

### Uncertainty analysis

The uncertainty analysis results indicate which parameters contribute the most to uncertainty in model outputs given their variances. The results are summarized in Table 3 and shows which parameters contribute the most to uncertainty in the merozoite and iRBC populations in all four models.

**Table 3:** Uncertainty analysis results in $\%$ contribution

	Merozoites		iRBCs	
Anderson	$\mu _P$	$k_1$	$\mu _P$	$k_1$
	42.0	13.2	48.3	15.2
Li	$\mu _P$	$k_1$	$\mu _P$	$k_1$
	45.4	32.2	48.1	34.1
Niger	$\mu _P$	$k_1$	$\mu _P$	$k_1$
	34.3	14.4	55.8	23.4
Okrinya	*r*	$\lambda _X$	$\lambda _X$	$\mu _Y$
	36.8	17.6	56.7	9.37

The table indicates which parameters contribute the most to uncertainty in the model outputs of merozoites and iRBCs. $\mu _P$—Death rate of immune effectors, $k_1$—Proliferation rate of immune effectors by iRBCs, *r*—Number of merozoites released per bursting iRBC, $\lambda _X$—Birth rate of healthy RBCs, $\mu _Y$—Natural death rate of iRBCs

For the first three models, parameters related to the death rate of immune effectors, $\mu _P$, and the proliferation rate of immune effectors due to the activation by iRBCs, $k_1$, contribute the most to uncertainty in merozoites and iRBCs model outputs. These parameters have a larger combined total contribution in the Li model as the total percentage adds to more than $70\%$, while it is considerably lower in other models. This indicates that there are other parameters in the Anderson and Niger models that can contribute significantly to uncertainty in variable outputs. $\mu _P$ and $k_1$ are significant contributors to uncertainty as variance in these parameters can greatly influence the strength of the immune system’s response to infection. In the Okrinya model the number of merozoites released per bursting iRBC and the birth rate of healthy RBCs lead to the most uncertainty in the merozoite population. The more merozoites released per bursting iRBC, the higher the infection and successive infections, leading to large changes in the model outputs. Changes in the birth rate of healthy RBCs ($\lambda _X$), lead to changes in the number of RBCs for infection, also leading to significant changes in the model outputs. The death rate of iRBCs ($\mu _Y$) similarly has a significant effect due to its impact on parasite production and immune response.

Overall, for three of the four models, the death rate of immune cells and their proliferation rate due to iRBCs have the largest contributions to uncertainty in model outputs. This indicates that the immune system components and lack of detail, contribute the most to uncertainty in the outputs of disease variables.

### Local sensitivity analysis

**Fig. 3:**
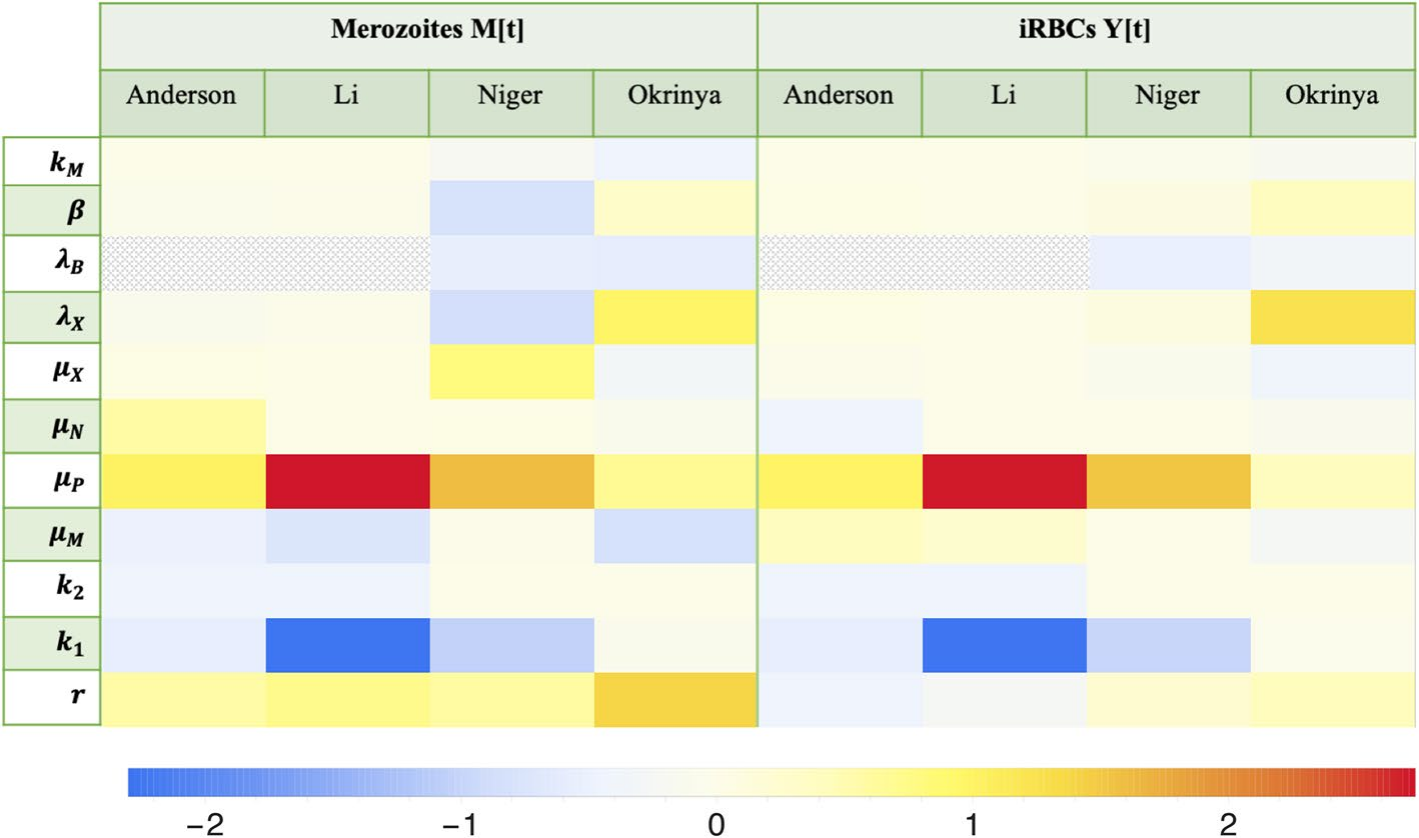
Results of the local sensitivity analysis. Parameters shown affect the disease variables the most in the reference state. Dark blue indicates a parameter that has a large negative influence on a variable, whereas dark red indicates a large positive influence on a variable. $\varvec{k_M}$—Elimination rate of merozoites by immune effectors, $\varvec{\beta }$—Probability of infection of RBCs with free roaming merozoites, $\varvec{\lambda _B}$—Production rate of immune cells, $\varvec{\lambda _X}$—Birth rate of healthy RBCs, $\varvec{\mu _X}$—Natural death rate of RBCs, $\varvec{\mu _N}$—Natural death rate of iRBCs, $\varvec{\mu _P}$—Death rate of innate immune cells/ immune effectors, $\varvec{\mu _M}$—Natural death rate of merozoites, $\varvec{k_2}$—Proliferation rate of immune effectors by merozoites, $\varvec{k_1}$—Proliferation rate of immune effectors by iRBCs, ${\varvec{r}}$—Number of merozoites released per bursting iRBC. Cross-hatching indicates parameters that were not present in a model

The local sensitivity analysis results concerning comparable parameters between the four models is shown as a heatmap in Fig. 3. Only the disease variables are indicated as it is these variables that are of clinical interest. The results indicate the percentage change in the value of a variable at steady state upon a $1\%$ change in a parameter value.

Four striking variable-parameter pairs are present in the Li model. The death rate of immune effectors, $\mu _P$, increases both disease variables the most. With a response coefficient larger than 2, the analysis illustrates that a $1\%$ increase in the death rate of immune effectors will increase the steady state population of merozoites and iRBCs by more than $2\%$. This is due to a higher death rate of immune cells leading to fewer active immune cells to fight the merozoites. The proliferation rate of immune effectors due to the activation by iRBCs ($k_1$) show the exact opposite, where a $1\%$ increase correlates with a decrease in the disease variable populations by more than $2\%$. This demonstrates that a small increase in how well the immune system’s response is activated, can lead to a dramatically better disease clearance. Additionally, the response coefficients obtained for the immune effector variables in the Li model showed extremely high responses on parameter perturbations, but was not included in the results shown here as it is a comparison of the disease variables only.

The death rate of immune effectors also delivers high response coefficients for the Anderson and Niger disease variables. These results emphasize the immune system’s response since maximizing the activation of the immune system relative to cell death assists with disease clearance. In the Okrinya model, the number of merozoites released per bursting iRBC, *r*, has the largest influence on the merozoite population, where the birth rate of healthy RBCs, $\lambda _X$, influences the iRBCs the most. Logically, the merozoite population will increase if more merozoites are released when a cell bursts and with a larger healthy RBC population, more cells can be infected to form iRBCs.

Interestingly, when inspecting the different models, the Okrinya model shows a direct correlation between the merozoite and iRBC populations, where if a parameter increases the population of merozoites, the iRBC population will also increase. This proportionality in the disease variable responses is also seen with most of the prominent results such as the responses with respect to $k_1$ and $\mu _P$ in all models. However, when considering the results for $\mu _M$ (the natural death rate of merozoites), it is evident that the merozoites decrease whereas the iRBC slightly increase in the models of Anderson and Li. One would expect that if the death rate of merozoites increases and the merozoite population decreases, there would be fewer merozoites to infect RBCs and thus fewer iRBCs. The same should hold for the number of merozoites released per iRBC (*r*), where an increase in the parameter increases the merozoites and the iRBCs in the Niger and Okrinya models. The counter-intuitive results in the Anderson and Li models lay bare the differences in the immune systems in the models, where small changes in infection close to the reference state are responded to differently.

Overall, the local sensitivity analysis revealed the greatest sensitivities for parameters that affect the immune system, the number of merozoites released by bursting iRBCs and the availability of healthy RBCs for infection. Of these processes, the death rate of immune cells and the proliferation rate of the immune effectors due to iRBCs have the largest effects on the disease variables.

### Local sensitivity robustness analysis

**Fig. 4:**
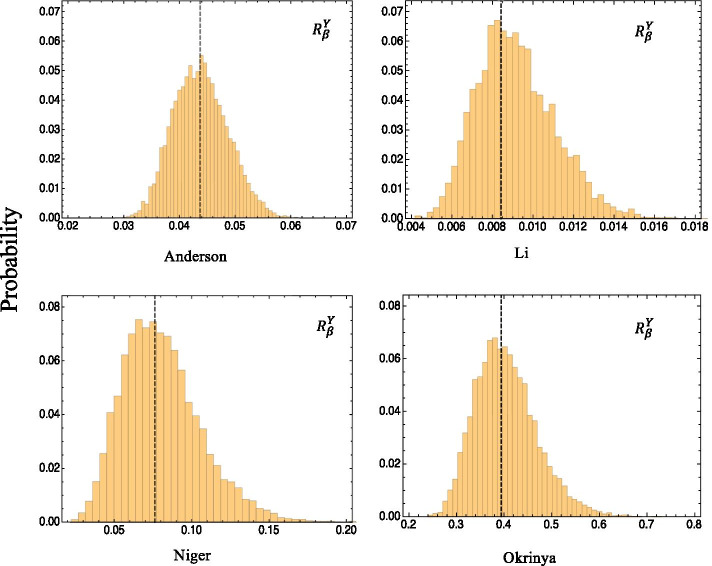
Local sensitivity robustness results. The histograms represent the pooled response coefficients for the sensitivity of the iRBCs, *Y*, for the probability of infection of RBCs with free roaming merozoites, $\beta$, obtained for all 10,000 parameter sets. The wild type response coefficients obtained with local sensitivity analysis is visualized as black dashed lines. All results shown here indicate good robustness of the local sensitivity results as the wild type response coefficients correspond to the most probable response coefficient in a population (the peak of the histogram)

Using the parameter sets from the robustness analysis, local sensitivity analysis was performed with respect to each parameter, with the model output generated from every parameter set used as a reference state. Response coefficients for a variable-parameter pair from all the parameter sets were pooled and visualized using probability distribution histograms. In Fig. 4, the same variable-parameter pair is displayed for all models. It illustrates the response coefficient distribution for the response of the iRBCs (*Y*) to the probability of infection of RBCs by free roaming merozoites. This variable-parameter pair was chosen for comparison, as it is also present in the local sensitivity analysis results section.

**Fig. 5:**
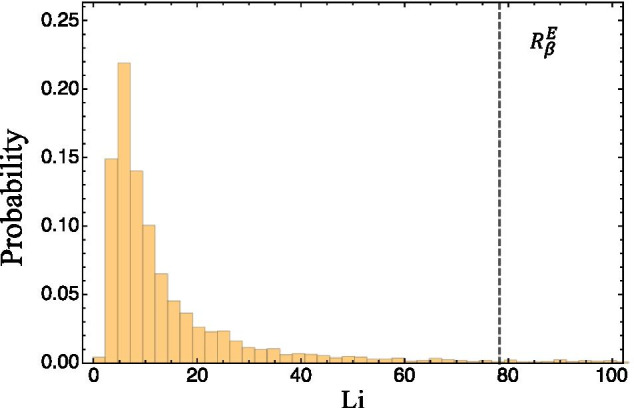
Local sensitivity robustness results for the Li model. The response coefficient distributions of the immune effectors’ population *E* for the probability of infection of RBCs with free roaming merozoites, $\beta$, is shown. The wild type response coefficient obtained with local sensitivity analysis is visualized as a black dashed line. Poor robustness of the local sensitivities is inferred as the wild type response coefficient does not correspond with the most probable response coefficient in a population (the peak of the histogram)

From the results it is evident that there is an approximately normal distribution for all four models. Furthermore, the response coefficients determined for all of the parameter sets are in a very small range, showing substantial conservation despite heterogeneity in a population setting. Results from the Anderson, Niger and Okrinya models displayed approximately normal distributions for all their variable-parameter pairs and their wild-type response coefficients correspond well with the most probable response in a population. The Li results, however, showed some irregularities with the immune effector variable pairs as can be seen in Fig. 5.

It is noteworthy how large the wild type response coefficients are for the Li model with a value of approximately 78, indicating that a $1\%$ change in the infection rate of healthy RBCs with merozoites, will increase the immune effector population by $78\%$. The results furthermore reveal the great difference between the wild type, indicated as the black dashed line, and most probable, the peak of the histogram, response coefficients. The wild-type response coefficients obtained with local sensitivity analysis showed large values for the immune effector *E* responses with respect to changes in most of the parameters. In Fig. 5 it is evident that the most probable response coefficient is much lower given these parameter ranges. Similar local sensitivity robustness behaviour was observed for all parameters that lead to large responses in the immune system variables in the local sensitivity analysis results. It should be noted, however, that the authors investigated the stability of their model and determined bifurcation parameters that produced oscillations. An example of one of the parameters used is $k_1$, where a value of $4.5001\times 10^{-5}$ resulted in stable steady state values. As this value increases to larger than $4.5045\times 10^{-5}$, a periodic solution appears [22]. These values show how a very small perturbation in some parameters could lead to a large alteration in the model behaviour. Although our analysis always considers the steady state value (or unstable steady state value in the case of oscillations), different parameter sets could lead to greatly varying response coefficients.

Overall, these results suggest that Anderson, Niger and Okrinya models all display conserved sensitivities and responses when considering population heterogeneity, whilst caution should be taken when interpreting analysis results of the Li model.

## Discussion

Models describing the immune response to malaria infection were analysed to study the differences in model formalism and sensitivity to parameter values. The methods used in this investigation can indicate when models are more relevant for describing disease dynamics within an individual as well as in a population, whilst also giving insight into the necessity for parameter value certainty.

Robustness analysis was performed to obtain an indication of the spread of outcomes in a population and how it compares to the wild type outcome. Results indicated that the Anderson model was the most robust of all the models showing the most resistance to changes in parameter values and yielding results showing the least variation in outputs. However, this model is the oldest of the models, with limited detail of the interactions between the host immune system and the malaria parasites. The second and third most robust models are those of Okrinya and Niger. Between the two, the Okrinya is more mechanistically descriptive of the within-host dynamics of malaria infection. Robustness as defined here should, however, be considered in the context of parameter distributions observed in a population since one would expect the model to display a range of predictions that correlate to what is observed in reality given such distributions.

Uncertainty analysis can be helpful to determine which parameters have a large influence on the uncertainty in model outputs. Parameter values were all varied within $10\%$ around their wild type values for analysis. However, the parameter variances can be much larger in reality, owing to the difficulty of estimating parameter values, as well as variations between individuals due to differences in factors such as diets, ages etc. Gaining more information on these parameters and their possible variances in a population could consequently be useful for further analysis and interpretations. Uncertainty analysis demonstrates how necessary it is to determine precise values or ranges for some parameters. In this study it was found that parameters of the immune system contributed the most to uncertainty in model outputs.

Local sensitivity analysis indicated that the death rate of immune effectors, the number of merozoites released per bursting iRBC and the proliferation of immune cells due to the presence of iRBC have the largest influence on the disease variables in all models around the published reference state. The Okrinya model further indicated the birth rate of healthy RBCs as a parameter of interest. We also found that a larger ratio of proliferation to death rate of the immune cells can drastically affect the model outcomes and help with the attack on infection, and moreover it seems to be the largest influencer in all the models. Combined with decreasing the number of merozoites released per bursting iRBC, this could lead to disease clearance. The two parameters corresponding to the immune system were, however, also present in most of the models as the parameters that contributed the most to uncertainty in model outputs.

Local sensitivity robustness analysis demonstrated how well response coefficients are conserved over the multi-dimensional parameter space. Good robustness of local sensitivities was achieved for most response coefficients in the models, i.e. the wild-type responses were well conserved in a heterogeneous population. The results of the Li model indicated poor conservation as the large response coefficients observed in local sensitivity analysis did not correspond with the much lower most probable response coefficients. Furthermore, in the Li model the range of some coefficients in a population varied from just above zero to approximately 80, showing the wide range of possible, but intuitively implausible, response coefficients in a population. The results for the Li model thus emphasises the necessity of this analysis, as it demonstrates how local sensitivity analysis may fail in giving insight into the responses in a population of different individuals. As is the case for this model, it could be as a result of transitions in qualitative behaviour such as the switch from steady states, to limit cycle oscillations observed here.

## Conclusion

The results from local sensitivity analysis emphasize the importance of the immune response on the disease dynamics of malaria infection, as well as highlight parameters like the death and proliferation rates of immune effectors that could be investigated for disease eradication as these have the largest effect on disease variables. To further establish trust in reliability and fidelity of model predictions the uncertainty in specific parameter values needs to be minimised and clinical ranges obtained where possible. The robustness analysis indicated that the Niger and Okrinya models were robust in the sense that plausible outputs were obtained close to the reference state and local sensitivity robustness analysis showed that the local sensitivity results are relatively well conserved in a population.

In the current study the goal was to establish a measure of confidence in models that encapsulate the core dynamics of a disease state, but the analyses described here can easily be extended to any deterministic models that have similar ODE structures, for malaria and for other diseases. For example, an improved within-host model could be constructed and analysed with a combination of components from the Niger and Okrinya models where models such as that from Song et al. [55] can be incorporated to include variables of resistant and non-resistant parasites as well as treatment parameters.

Although not the focus of this study, an additional benefit of sensitivity analysis as conducted here is that it can also point to possible drug targets and whether these targets are conserved in a population. Local sensitivity analysis indicated that the number of merozoites released per bursting iRBC has a large effect on all models and that an increased ratio of immune effector proliferation to death rate could be beneficial to disease clearance. The conservation of these results in the local sensitivity robustness analysis shows that this could hold true in a population as well.

## Methods of analysis

All reproductions and analyses of the models were completed in Wolfram Mathematica version 12 using standard Mathematica functions. Code used for analyses is provided as executable notebooks in Additional files 2–5, and in pdf format in Additional file 6.

### Robustness analysis

Robustness analysis was used to determine the ranges and distributions of the disease variable outputs of each model. This method of analysis can indicate the expected steady state values of within-host variables in a population and can also show which models are the most resilient to changes in model inputs (i.e. parameters). Monte Carlo (MC) random sampling from uniform distributions was therefore used to obtain 10,000 parameter sets. In the absence of known ranges of parameters and to facilitate direct model output comparisons, all parameters were varied simultaneously within a $10\%$ range of their wild type values for all models. These sets mimic different parameter sets of individuals within a population. All sets were used to determine the distribution of the disease variables’ values at steady state. Three different disease states were simulated to determine the difference in the ranges of the steady state values for the RBC and iRBC populations: (i) no infection, (ii) infection without an immune response and, (iii) infection with an immune response. Results for two of the models are shown in the results section for comparison. The sub-models where the immune system is incorporated from each of the four publications were then analysed with regards to their disease variables in the endemic state. Where required for comparison between models, in silico datasets were normalized to their median. The results are visualized with box-and-whisker plots.

### Uncertainty analysis

Here we followed the method set out in [56]. To determine the uncertainty in variable outputs due to variability in parameter values, the variance ($\sigma ^2$) of the natural logarithm of each parameter ($p_j$) is first calculated:

$$\begin{aligned} \sigma ^2(\ln {p_j}) \end{aligned}$$

However, as the variance of many of the parameters in these biological models are not known, the variance of each parameter was calculated using a uniform distribution around its wild-type value with upper and lower bounds given by wild-type value $\pm 10\%$. The results therefore indicate which parameters contributes the most to uncertainty in the model variable outputs. The individual contribution of each parameter variance $\sigma ^2_j(\ln {p_j})$ to the total variance of each variable $\sigma _j^2 (V_i )$ is then calculated using:

$$\begin{aligned} \sigma ^2_j(V_i) = \sigma ^2(\ln {p_j})\left( \frac{\partial {V_i}}{\partial {\ln }{p_j}}\right) ^2 \end{aligned}$$

The total variance of each variable can be determined by summation of the individual contributions:

$$\begin{aligned} \sigma ^2(V_j) =\sum _j \sigma _j^2 (V_i) \end{aligned}$$

The contribution of each parameter to model variable uncertainty is then given by:

$$\begin{aligned} \%\text {uc}_{ij} = \frac{\sigma _j^2(V_i)}{\sigma ^2 (V_i)} \times 100 \end{aligned}$$

### Local sensitivity analysis

To determine for which parameters the disease variables are most sensitive in the reference state, local sensitivity analysis was performed . The method entails the perturbation of one parameter at a time to see the effect on model outputs, indicated as a response coefficient. A response coefficient describes the percentage change of a model output — in this case the steady state values of different variables *V *— upon a $1\%$ change in model inputs or parameters *p*:

$$\begin{aligned} R_p^V = \frac{\partial {V}}{\partial {p}} \times \frac{p}{V} \end{aligned}$$

Numerically, the derivative is approximated by a finite difference formula using small perturbations around the wild type parameter value and noting the change on model output.

### Local sensitivity robustness analysis

Local sensitivity robustness analysis was used to test for conservation of the local sensitivity analysis results given parameter variations in a population. It would therefore be an indicator of whether the response coefficient results of a parameter-variable pair in the individual with the wild type parameter set, is the most common result in a population with differing parameter sets. Local sensitivity analysis, as described above, was therefore performed for 10,000 parameter sets obtained using MC random sampling from the uniform distributions of parameters in the complete parameter space (see “Robustness analysis” above). The response coefficients for a given parameter was pooled from each set and histograms were used to visualize results.

## Additional file

**Additional file 1.** Model parameters and variables.

**Additional file 2.** Anderson analysis (Mathematica notebook).

**Additional file 3.** Li analysis (Mathematica notebook).

**Additional file 4.** Niger analysis (Mathematica notebook).

**Additional file 5.** Okrinya analysis (Mathematica notebook).

**Additional file 6.** Analysis code for all models.

## Data Availability

All models analysed in this study are available in the curated database of JWS Online: https://jjj.bio.vu.nl/models/anderson1, https://jjj.bio.vu.nl/models/niger1, https://jjj.bio.vu.nl/models/li2, https://jjj.bio.vu.nl/models/okrinya1 Model parameter values and variable definitions are also provided in Additional file 1. Code used for analyses is provided as executable notebooks in Additional files 2–5, and in pdf format in Additional file 6.

## Abbreviations

RBC: Red blood cell
iRBC: Infected red blood cell
MCA: Metabolic control analysis
MC: Monte Carlo
