# Supplementary material for: Uncovering the effects of heterogeneity and parameter sensitivity on within-host dynamics of disease: malaria as a case study
Source: BMC Bioinformatics. 2021 Jul 24;22:384. doi: 10.1186/s12859-021-04289-z (PMC8305899; doi:10.1186/s12859-021-04289-z)
Supplement: Supplementary file 1 — Additional file 1. Model parameters and variables. [file 12859_2021_4289_MOESM1_ESM.pdf]

Additional file 1: "Uncovering the effects of heterogeneity and parameter sensitivity on within-host dynamics of disease: malaria as a case study"

Model parameters and variables

The parameter definitions and values are shown in Tables 1 - 4 below. In the case where multiple models were published, only the relevant values used for analysis are included in these tables.

Table 1 Parameter and initial values for Anderson et al. [1]

Parameter	Description	Value	Unit
λ	Recruitment rate of healthy RBCs	1	$/day$
μ	Natural death rate of RBCs	0.00833	$/day$
β	Probability of infection of RBCs with free roaming merozoites	0.1	$/day$
α	Death rate of iRBCs	0.2	$/day$
r	Merozoites released per bursting iRBC	16	$/day$
d	Natural death rate of free roaming merozoites	72	$/day$
h	Rate of antibody-mediated killing of free roaming merozoites	0.1	$/day$
γ	Proliferation rate of T-lymphocytes due to merozoites	1.0	$/day$
a	Natural death rate of T-lymphocytes	0.05	$/day$
g	Rate of cytotoxic killing of iRBCs	0.05	$/day$
k	Proliferation rate of T-lymphocytes due to iRBCs	0.05	$/day$
$x(0)$	Initial density of healthy RBCs	120	−
$y(0)$	Initial density of iRBCs	0	−
$s(0)$	Initial density of merozoites after infection	1	−
$T(0)$	Initial density of T-lymphocytes	0.0001	−

Table 2 Parameter and initial values for Li et al. [2]. The endemic equilibrium state of disease was chosen from the published models.

Parameter	Description	Value	Unit
λ	Production rate of RBCs	4.15×10^4	$cells/\mu l/day$
d_1	Decay rate of RBCs	8.3×10^{-3}	$/day$
μ	Decay rate of malaria parasites	48	$/day$
d_2	Decay rate of immune effectors	0.04	$/day$
α	Infection rate of RBCs by malaria parasites	9×10^{-7}	$\mu l/cell/day$
δ	Decay rate of iRBCs	1.0	$/day$
r	Product rate of malaria parasites	12	$/day$
p_1	Removal rate of iRBCs by immune system	10^{-8}	$\mu l/cell/day$
p_2	Removal rate of malaria parasites by immune system	10^{-8}	$\mu l/cell/day$
k_1	Proliferation rate of immune effectors by iRBCs	2.5×10^{-5}	$\mu l/cell/day$
k_2	Proliferation rate of immune effectors by merozoites	1.03×10^{-5}	$\mu l/cell/day$
β	$\frac{1}{\beta}$ half saturation constant for iRBCs	5×10^{-4}	$\mu l/cell$
γ	$\frac{1}{\gamma}$ half saturation constant for malaria parasite	6.67×10^{-4}	$\mu l/cell$
$H(0)$	Initial population of RBCs	5×10^6	$cells/\mu l$
$I(0)$	Initial population of iRBCs	0	$cells/\mu l$
$M(0)$	Initial population of malaria parasites	10^4	$cells/\mu l$
$E(0)$	Initial population of immune effectors	10^{-4}	$cells/\mu l$

Table 3 Parameter and initial values for Niger et al. [3]. Initial values may vary and parameter values were published without units.

Parameter	Description	Value	Unit
λ_X	Production rate of RBCs from the bone marrow	41664	$-$
λ_B	Production rate of immune cells	30	$-$
μ_X	Natural death rate of uninfected RBCs	0.8	$-$
$\mu_{Y_i}(i=1-4)$	Natural death rate of iRBCs	0.5	$-$
μ_{Y_5}	Natural death rate of iRBCs	1.0	$-$
μ_M	Natural death rate of merozoites	3.0	$-$
μ_B	Death rate of immune cells	1.53	$-$
μ_A	Deterioration rate of antibodies	0.4	$-$
μ	Loss of merozoites due to infection of RBCs	1	$-$
β	Rate of infection	8×10^{-4}	$-$
$\gamma_i(i=1-5)$	Progression rate of iRBCs from Stage (i) to Stage $(i+1)$	1.5	$-$
$k_i(i=1-5)$	Immunosensitivity of iRBCs	0.01	$-$
k_M	Immunosensitivity of merozoites	0.3	$-$
ρ_1	Immunogenicity of iRBCs and merozoites	0.001	$-$
$\rho_i(i=2-6)$	Immunogenicity of iRBCs and merozoites	10^{-5}	$-$
η	Maximum rate of increase in antibodies	0.6	$-$
r	Number of merozoites	16	$-$
$X(0)$	Initial concentration of healthy RBCS	500	$-$
$Y_1(0)$	Initial concentration of iRBCs	$-$	$-$
$M(0)$	Initial concentration of merozoites	10	$-$
$B(0)$	Initial concentration of immune cells	$-$	$-$
$A(0)$	Initial concentration of antibodies	$-$	$-$

Table 4 Parameter and initial values for Okrinya et al. [4]

Parameter	Description	Value	Unit
λ_x	Rate at which RBCs are recruited	4.15×10^4	$cells/\mu l/day$
β_x	Rate constant for infection rate of RBCs	$4.9{\times}10^{-6}$	$\mu l/cell/day$
μ_x	Natural per capita death rate of RBCs	0.0083	$/day$
μ_n	Natural death rate of iRBCs	0.055	$/day$
μ_y	Conversion rate of iRBCs to merozoites	0.5	$/day$
r	Number of merozoites release per bursting schizont	16	$-$
μ_m	Death rate of merozoites	48	$/day$
θ	Fraction of merozoites converting to gametocytes	$6.4{\times}10^{-3}$	$-$
μ_g	Natural per capita death rate of gametocytes	0.02	$/day$
c_0	Efficiency of antibodies in blocking merozoite invasion	0.6	$cell/mol$
c_1	Efficiency of antibodies in blocking merozoite release	1.12	$cell/mol$
k_a	Antibody induced Fc-dependent killing rate of iRBCs	1.38	$cell/mol$
k_b	Antibody induced Fc-dependent killing rate of merozoites	12	$cell/mol$
k_c	Antibody induced Fc-dependent killing rate of gametocytes	1.26	$cell/mol$
k_y	Elimination rate of iRBCs by innate immune cells	0.9	$\mu l/cell/day$
k_m	Elimination rate of merozoites by innate immune cells	1.18×10^2	$\mu l/cell/day$
k_g	Elimination rate of gametocytes by innate immune cells	1.4	$\mu l/cell/day$
b_m	Supply rate of immune cells from stem cells	0.038	$cell/\mu l/day$
η_1	Parasite induced innate immune cell production rate	$1.3{\times}10^{-7}$	$/day$
η_2	Parasite induced spesific immune cell production rate	3×10^{-7}	mol $\mu l/cell^2/day$
ϕ	Phagocyte growth difference between merozoites and iRBCs	2	$-$
g_2	Antibody production difference between merozoites and iRBCs	0.85	$-$
k_d	Deterioration rate of innate immune cells due to iRBC killing	$2.8{\times}10^{-9}$	$\mu l/cell/day$
k_n	Deterioration rate of innate immune cells due to interaction with merozoites	$3.1{\times}10^{-9}$	$\mu l/cell/day$
η_3	Deterioration rate of antibodies due to interaction with iRBCs	$4.5{\times}10^{-8}$	$\mu l/cell/day$
η_4	Deterioration rate of antibodies due to interaction with merozoites	$3.4{\times}10^{-8}$	$\mu l/cell/day$
μ_p	Death rate of innate immune cells	0.3	$/day$
A_0	Starting density of antibodies	0	$moll/cell$
μ_A	Death rate of antibodies	0.3	$/day$
$X(0)$	Initial concentration of RBCs	5×10^6	$cell/\mu l$
$Y(0)$	Initial concentration of iRBCs	0	$cell/\mu l$
$M(0)$	Initial concentration of merozoites	0.105	$cell/\mu l$
$G(0)$	Initial concentration of gametocytes	0	$cell/\mu l$
$P(0)$	Initial concentration of innate immune cells	0.127	$cell/\mu l$
$A(0)$	Initial concentration of antibodies	0	$mol/cell$

Author details

References

1. Anderson RM, May RM, Gupta S. Non-linear phenomena in host-parasite interactions. Parasitology. 1989;99(S1):S59–S79.
2. Li Y, Ruan S, Xiao D. The within-host dynamics of malaria infection with immune response. Mathematical Biosciences and Engineering. 2011;8(4):999–1018.
3. Niger AM, Gumel AB. Immune response and imperfect vaccine in malaria dynamics. Mathematical Population Studies. 2011;18(2):55–86.
4. Okrinya AB. Mathematical Modelling of Malaria Transmission and Pathogenesis. Loughborough University; 2014.
