# Supplementary material for: Uncovering the effects of heterogeneity and parameter sensitivity on within-host dynamics of disease: malaria as a case study
Source: BMC Bioinformatics. 2021 Jul 24;22:384. doi: 10.1186/s12859-021-04289-z (PMC8305899; doi:10.1186/s12859-021-04289-z)
Supplement: Supplementary file 6 — Additional file 6. Analysis code for all models. [file 12859_2021_4289_MOESM6_ESM.pdf]

Additional file 6: "Uncovering the effects of heterogeneity and parameter sensitivity on within-host dynamics of disease: malaria as a case study"

Analysis code

Additional file 2 : "Uncovering the effects of heterogeneity and parameter sensitivity on within-host dynamics of disease: malaria as a case study"

Anderson Analysis

In[•]:=
```
(*Helper functions defined for steady state
  determination and parameter modifications*)
ststSolMatFormR[model_, time_ : 100, startingpoint_ : {}] :=
  Block[{odes, rateEquations, assignments, parameters,
    initialValues, variables, timeCourse, findRootEquations,
    findRootVariables, steadyStateVariables, fluxes},
   odes = Thread[D[model[[1, 1]], t] == model[[1, 2]].model[[1, 3]]];
   rateEquations = model[[2]];
   assignments = model[[5]];
   parameters = model[[4]];
   initialValues = model[[3]];
   variables = model[[1, 1]];

   (*Steady-state solution initialized with result of time evolution*)
   findRootEquations = odes /. D[_[t], t] → 0;
findRootVariables = If[startingpoint == {},
     timeCourse = NDSolve[Join[odes, initialValues] //. rateEquations //.
         assignments //. parameters, variables, {t, 0, time},
       (*Method→{"StiffnessSwitching","NonstiffTest"→"NormBound"}*)
       MaxSteps → Infinity, Method → "BDF", AccuracyGoal → 10,
       PrecisionGoal → 10, MaxStepSize → time / 1000.];
     Partition[Flatten[{#, # /. timeCourse /. t → time} & /@ variables], 2],
     Partition[Flatten[{#[[1]], #[[2]]} & /@ startingpoint[[1]]], 2]];
steadyStateVariables = FindRoot[findRootEquations //. rateEquations //.
       assignments //. parameters, findRootVariables, MaxIterations → 1000];
fluxes = # //. assignments //. parameters /. steadyStateVariables & /@
     rateEquations;
   {steadyStateVariables, fluxes}
  ];

addParametersToModel[model_, par_] :=
  Block[{parameters, newParameters, newModel, unmodified},
   parameters = model[[4]];
   newParameters = Flatten[{par}][[ ;; , 1]];
   unmodified = Select[parameters, MemberQ[newParameters, #[[1]]] == False &];
   newParameters = Join[par, unmodified];
   newModel = model;
   newModel[[4]] = newParameters;
   newModel];
```

In[•]:=
```
(*Model imported from JWS Online*)
modeldMF =
  ToExpression[ URLExecute["https://jjj.bio.vu.nl/models/anderson1/mf"]];;
```

In[•]:=
```
(*Local Sensitivity Analysis*)
model = modeldMF;
oldStSt = ststSolMatFormR[model, 100 000];
oldStStD = oldStSt;
vars = Select[oldStSt[[1]], #[[2]] > 0.0001 &][[ ;; , 1]];
respTabD = Table[
  param = model[[4, j]];
  tempMod = addParametersToModel[model, {param[[1]] → param[[2]] * 1.00001}];
  newStStUp = ststSolMatFormR[tempMod, 100 000];
  tempMod = addParametersToModel[model, {param[[1]] → param[[2]] * 0.99999}];
  newStStDown = ststSolMatFormR[tempMod, 100 000];
  respTab = Table[{param[[1]], vars[[i]],
     ((vars[[i]] /. newStStUp[[1]]) - (vars[[i]] /. newStStDown[[1]])) /
      (2 * 0.00001 vars[[i]] /. oldStSt[[1]])}, {i, 1, Length[vars]}];
  respTab, {j, 1, Length[model[[4]]]}];
validPairsD = Select[Flatten[respTabD, 1], ! #[[3]] === 0. &][[ ;; , {1, 2}]];
```

In[•]:=
```
respTabD
```

Out[•]=
```
{{{model`a, model`S[t], 1.01473}, {model`a, model`T[t], -0.401403},
  {model`a, model`X[t], -0.211555}, {model`a, model`Y[t], 0.988484}},
 {{model`alfa, model`S[t], 0.567077}, {model`alfa, model`T[t], 0.76668},
  {model`alfa, model`X[t], -0.118227}, {model`alfa, model`Y[t], -0.443435}},
 {{model`beta, model`S[t], -0.055912}, {model`beta, model`T[t], 1.52394},
  {model`beta, model`X[t], -0.196827}, {model`beta, model`Y[t], 0.0437213}},
 {{model`d, model`S[t], -0.496438}, {model`d, model`T[t], -1.69202},
  {model`d, model`X[t], 0.1035}, {model`d, model`Y[t], 0.388198}},
 {{model`g, model`S[t], 0.00236514}, {model`g, model`T[t], -0.991939},
  {model`g, model`X[t], -0.000493093}, {model`g, model`Y[t], -0.00184946}},
 {{model`gamma, model`S[t], -0.445285}, {model`gamma, model`T[t], 0.176144},
  {model`gamma, model`X[t], 0.0928348}, {model`gamma, model`Y[t], -0.433768}},
 {{model`h, model`S[t], -0.00236514}, {model`h, model`T[t], -0.00806115},
  {model`h, model`X[t], 0.000493093}, {model`h, model`Y[t], 0.00184946}},
 {{model`k, model`S[t], -0.569443}, {model`k, model`T[t], 0.225259},
  {model`k, model`X[t], 0.11872}, {model`k, model`Y[t], -0.554715}},
 {{model`lambda, model`S[t], -0.0706391}, {model`lambda, model`T[t], 1.92534},
  {model`lambda, model`X[t], 1.01473}, {model`lambda, model`Y[t], 0.0552374}},
 {{model`mu, model`S[t], 0.055912}, {model`mu, model`T[t], -1.52394},
  {model`mu, model`X[t], -0.803173}, {model`mu, model`Y[t], -0.0437213}},
 {{model`r, model`S[t], 0.564319}, {model`r, model`T[t], 1.92338},
  {model`r, model`X[t], -0.117652}, {model`r, model`Y[t], -0.441278}},
 {{model`default, model`S[t], 0.}, {model`default, model`T[t], 0.},
  {model`default, model`X[t], 0.}, {model`default, model`Y[t], 0.}}}
```

In[•]:=
```
(*Monte Carlo Random sampling and Robustness analysis*)
model = modeldMF;
pramD = modeldMF[[4]];
startingpoint = Table[#[[1]] → #[[2]] & /@ oldStStD[[i]], {i, 1, 2}];
pramnamesD = pramD[[ ;; , 1]];
pramvaluesD = pramD[[ ;; , 2]];
pramsizeD = Length[pramnamesD];
noOfParamsetsD = 10 000;
paramSetsMC = ParallelTable[Table[
    pramnamesD[[i]] → RandomReal[{0.9 pramvaluesD[[i]], 1.1 pramvaluesD[[i]]}],
    {i, 1, Length[pramnamesD]}], {z, 1, noOfParamsetsD}];
paramSetsMCd = paramSetsMC;
ststD = ParallelTable[
   {Quiet[ststSolMatFormR[
      addParametersToModel[model, paramSetsMCd[[i]]], 100 000, startingpoint]],
    paramSetsMCd[[i]]}, {i, 1, Length[paramSetsMCd]}];
validStStD = Select[ststD, (model`X[t] /. #[[1, 1]]) > 0.00001 &&
     (model`Y[t] /. #[[1, 1]]) > 0.00001 &];
XvalsD = model`X[t] /. validStStD[[ ;; , 1, 1]];
YvalsD = model`Y[t] /. validStStD[[ ;; , 1, 1]];
SvalsD = model`S[t] /. validStStD[[ ;; , 1, 1]];
TvalsD = model`T[t] /. validStStD[[ ;; , 1, 1]];
```

In[•]:=
```
(*To Export each variable's achieved steady states for each parameter set*)
(*Export[NotebookDirectory[]<>"AndersonXss.txt",XvalsD];
Export[NotebookDirectory[]<>"AndersonYss.txt",YvalsD];
Export[NotebookDirectory[]<>"AndersonSss.txt",SvalsD];
Export[NotebookDirectory[]<>"AndersonTss.txt",TvalsD];*)
```

In[•]:=
```
(*A box–and–whisker plot showing Robustness of iRBCs*)
YvalsDmed = YvalsD / Median[YvalsD];
bwplotY = BoxWhiskerChart[{YvalsDmed}, FrameLabel → {"", "iRBC"},
   ChartStyle → {Red}, LabelStyle → {FontSize → 16, FontFamily → "Helvetica"},
   FrameStyle → Thickness[0.003],
   BaseStyle → {FontSize → 20, FontFamily → "Helvetica"}, ImageSize → 500,
   AspectRatio → 1 / 1.5(*,PlotRange→{All,{0.4,2.1}}*)]
```

Out[•]=
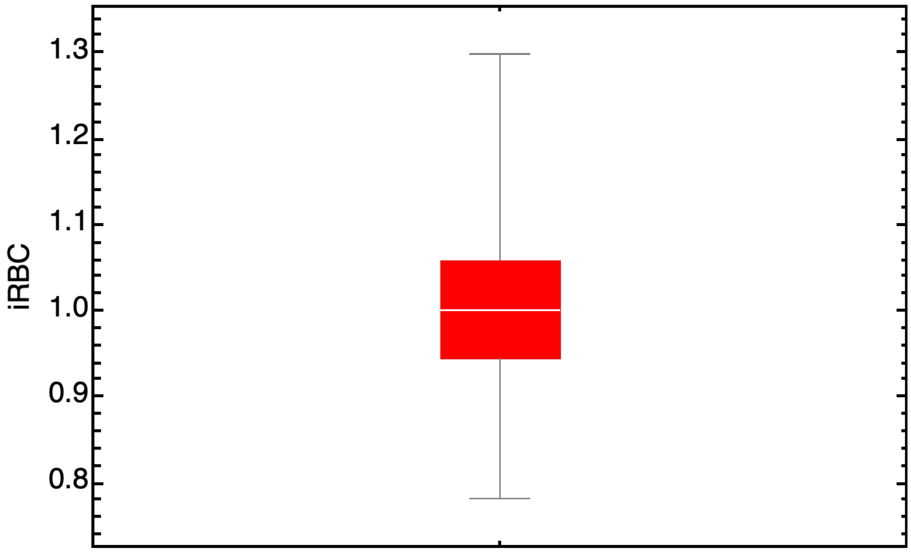

In[◦]:=
```
(*Robustness analysis of LSA*)
model = modeldMF;
validStSt = validStStD;
oldStSt = (*ststSolMatFormR[model,100000]*)oldStStD;
vars = Select[oldStSt[[1]], #[[2]] > 0.001 &][[ ;; , 1]];
parameterSpaceResponsesModelD = ParallelTable[
   paramSet = validStSt[[z, 2]];
   oldStSt = validStSt[[z, 1]];
   startingpoint = Table[#[[1]] → #[[2]] & /@ oldStSt[[i]], {i, 1, 2}];
   model = addParametersToModel[modeldMF, paramSet];
   paramSetResp = Table[
     param = paramSet[[j]];
     tempMod =
      addParametersToModel[model, {param[[1]] → param[[2]] * (*1.001*) 1.001}];
     newStStUp = Quiet[ststSolMatFormR[tempMod, 10 000, startingpoint]];
     tempMod =
      addParametersToModel[model, {param[[1]] → param[[2]] * (*0.999*) 0.999}];
     newStStDown = Quiet[ststSolMatFormR[tempMod, 10 000, startingpoint]];
     respTab = Table[
       (*If[(vars[[i]]/.newStStUp[[1]])<1||(vars[[i]]/.newStStDown[[1]])<1,
        {param[[1]],vars[[i]],Null},*)
       {param[[1]], vars[[i]], ((vars[[i]] /. newStStUp[[1]]) -
            (vars[[i]] /. newStStDown[[1]])) /
          (2 * 0.001 vars[[i]] /. oldStSt[[1]])}, {i, 1, Length[vars]}];
     respTab, {j, 1, Length[model[[4]]]}];
   {paramSetResp, paramSet},
   {z, 1, Length[validStSt]}];
parameterSpaceResponsesModelDFlat = Select[
   Flatten[parameterSpaceResponsesModelD[[ ;; , 1]], 2], ! #[[3]] === Null &];
```

In[◦]:=
```
validPairs = validPairsD;
respBigTabD = Table[
   paramName = model[[4, i, 1]];
   varName = vars[[j]];
   Select[parameterSpaceResponsesModelDFlat, #[[1]] === paramName &&
      #[[2]] === varName && MemberQ[validPairs, {paramName, varName}] == True &],
   {i, 1, Length[model[[4]]]}, {j, 1, Length[vars]}];
testTab = DeleteCases[Flatten[respBigTabD, 2], {}];
testTab2 = Partition[testTab, Length[validStStD]];
```

In[•]:=
```
(*Histograms*)
tablaDanderson = Table[
  parName = StringReplace[ToString[testTab2[[i, 1, 1]]], "model`" → ""];
  varName = StringReplace[ToString[testTab2[[i, 1, 2]]], "model`" → ""];
  wtResponse = Select[Flatten[respTabD, 1],
     {#[[1]], #[[2]]} == {testTab2[[i, 1, 1]], testTab2[[i, 1, 2]]} &][[1, 3]];
  Show[Histogram[testTab2[[i, ;; , 3]],
    {Min[testTab2[[i, ;; , 3]]], Max[testTab2[[i, ;; , 3]]],
     Abs[Max[testTab2[[i, ;; , 3]]] - Min[testTab2[[i, ;; , 3]]]] / 50},
    "Probability", Frame → True, FrameLabel → {R^varName_parName, "Probablity"},
    LabelStyle → {FontSize → 16, FontFamily → "Helvetica"},
    FrameStyle → Thickness[0.003], PlotRange → All,
    BaseStyle → {FontSize → 20, FontFamily → "Helvetica"}, ImageSize → 500,
    AspectRatio → 1 / 1.5, Axes → False, PlotRange → {{0, 0.1}, {0, 0.1}} ],
   ListLinePlot[{{wtResponse, 0}, {wtResponse, 1}},
    PlotStyle → Directive[Black, Opacity[0.7], Dashed, Thickness[0.005]]],
   PlotRange → {{0.02, 0.07}, {0, 0.07}}],
  {i, 12, 12(*Length[testTab2]*)}]
(*tablaD[[1]]*)
```

Out[•]= {

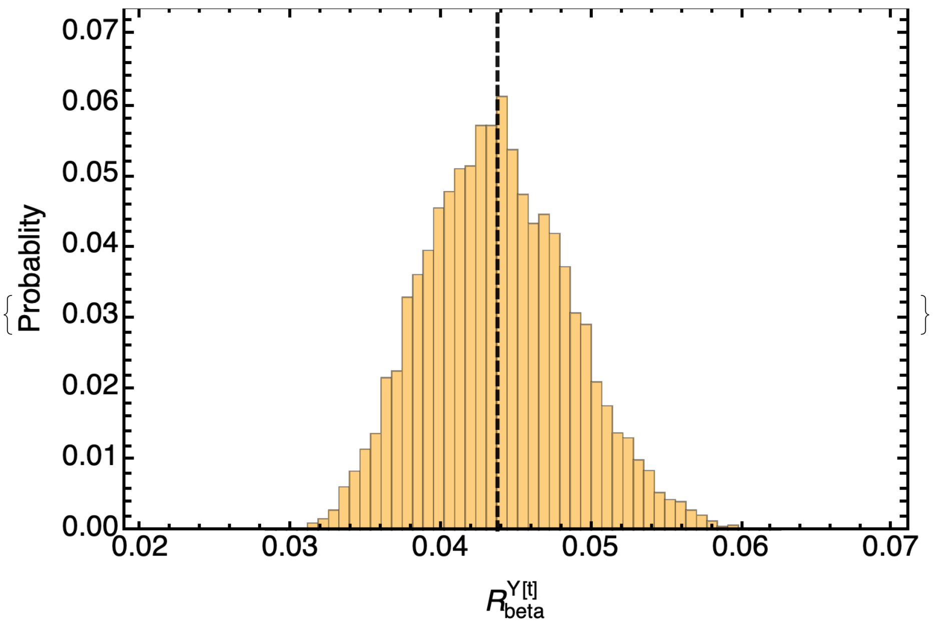

}

In[•]:=
```
(*Uncertainty analysis*)
```

In[•]:=
```
model = modeldMF;
oldStSt = ststSolMatFormR[model, 100 000];
vars = Select[oldStSt[[1]], #[[2]] > 0.0001 &][[ ;; , 1]];
oldStStD = oldStSt;
startingpoint = Table[#[[1]] → #[[2]] & /@ oldStStD[[i]], {i, 1, 2}];
paramsToScan = model[[4]];
paramNames = paramsToScan[[ ;; , 1]];
paramVals = paramsToScan[[ ;; , 2]];
paramLogExtremes = Table[
   var = 0.10;
   paramNames[[i]] → {(1 - var) * paramVals[[i]], (1 + var) * paramVals[[i]]},
   {i, 1, Length[paramNames]}];

paramLogVariance =
  #[[1]] → If[#[[2]] ≠ {0., 0.}, Variance[Log[RandomVariate[UniformDistribution[
          {#[[2, 1]], #[[2, 2]]}], 10 000 000]]], 0] & /@ paramLogExtremes;
(*(Integrate[(Log[x])^2./(#[[2,2]]-#[[2,1]]),{x,#[[2,1]],#[[2,2]]}]-
     Integrate[(Log[x])/(#[[2,2]]-#[[2,1]]),{x,#[[2,1]],#[[2,2]]}]^
      2)&/@paramLogExtremes;*)


respTabD = Flatten[Table[
    param = model[[4, j]];
    tempMod = addParametersToModel[model, {param[[1]] → param[[2]] * 1.0001}];
    newStStUp = ststSolMatFormR[tempMod, 10 000, startingpoint];
    tempMod = addParametersToModel[model, {param[[1]] → param[[2]] * 0.9999}];
    newStStDown = ststSolMatFormR[tempMod, 100 000, startingpoint];
    respTab = Table[{param[[1]], vars[[i]], (param[[1]] /. paramLogVariance) *
        (((vars[[i]] /. newStStUp[[1]]) - (vars[[i]] /. oldStSt[[1]])) /
           (0.0001)) ^2}, {i, 1, Length[vars]}];
    respTab, {j, 1, Length[model[[4]]]}], 1];

overallVariances =
  Thread[vars → (Total[#] & /@ Table[Select[respTabD, #[[2]] == vars[[i]] &&
           ! #[[3]] === Indeterminate &], {i, 1, Length[vars]}][[ ;; ,  ;; , 3]])];

contributionOfPars = Table[
   num = Flatten[
      Select[respTabD, #[[1]] == paramNames[[i]] && #[[2]] == vars[[j]] &]][[3]];
   denom = vars[[j]] /. overallVariances;
   {paramNames[[i]], vars[[j]], num / denom * 100},
   {j, 1, Length[vars]}, {i, 1, Length[paramNames]}];
TableForm[contributionOfPars]
```

Out[∘]//TableForm=

model\`a<br>model\`S[t]<br>42.0318	model\`alfa<br>model\`S[t]<br>13.1233	model\`beta<br>model\`S[t]<br>0.127609	model\`d<br>model\`S[t]<br>10.0594	model\`g<br>model\`S[t]<br>0.000228266	model\`gamma<br>model\`S[t]<br>8.09378
model\`a<br>model\`T[t]<br>0.963211	model\`alfa<br>model\`T[t]<br>3.51326	model\`beta<br>model\`T[t]<br>13.8835	model\`d<br>model\`T[t]<br>17.1127	model\`g<br>model\`T[t]<br>5.88024	model\`gamma<br>model\`T[t]<br>0.185489
model\`a<br>model\`X[t]<br>2.45976	model\`alfa<br>model\`X[t]<br>0.768008	model\`beta<br>model\`X[t]<br>2.12926	model\`d<br>model\`X[t]<br>0.588724	model\`g<br>model\`X[t]<br>0.000013359	model\`gamma<br>model\`X[t]<br>0.473688
model\`a<br>model\`Y[t]<br>48.3291	model\`alfa<br>model\`Y[t]<br>9.72323	model\`beta<br>model\`Y[t]<br>0.094547	model\`d<br>model\`Y[t]<br>7.45311	model\`g<br>model\`Y[t]<br>0.000169125	model\`gamma<br>model\`Y[t]<br>9.30646

In[∘]:=

```
NotebookSave[];
```

Additional file 3 : "Uncovering the effects of heterogeneity and parameter sensitivity on within-host dynamics of disease: malaria as a case study"

In[•]:=

Li Analysis

```
In[•]:= (*Helper functions defined for steady state
  determination and parameter modifications*)
ststSolMatFormR[model_, time_ : 100, startingpoint_ : {}] :=
  Block[{odes, rateEquations, assignments, parameters,
    initialValues, variables, timeCourse, findRootEquations,
    findRootVariables, steadyStateVariables, fluxes},
   odes = Thread[D[model[[1, 1]], t] == model[[1, 2]].model[[1, 3]]];
   rateEquations = model[[2]];
   assignments = model[[5]];
   parameters = model[[4]];
   initialValues = model[[3]];
   variables = model[[1, 1]];

   (*Steady-state solution initialized with result of time evolution*)
   findRootEquations = odes /. D[_[t], t] → 0;
findRootVariables = If[startingpoint == {},
     timeCourse = NDSolve[Join[odes, initialValues] //. rateEquations //.
         assignments //. parameters, variables, {t, 0, time},
       (*Method→{"StiffnessSwitching","NonstiffTest"→"NormBound"}*)
       MaxSteps → Infinity, Method → "BDF", AccuracyGoal → 10,
       PrecisionGoal → 10, MaxStepSize → time / 1000.];
     Partition[Flatten[{#, # /. timeCourse /. t → time} & /@ variables], 2],
     Partition[Flatten[{#[[1]], #[[2]]} & /@ startingpoint[[1]]], 2]];
steadyStateVariables = FindRoot[findRootEquations //. rateEquations //.
       assignments //. parameters, findRootVariables, MaxIterations → 1000];
fluxes = # //. assignments //. parameters /. steadyStateVariables & /@
     rateEquations;
   {steadyStateVariables, fluxes}
  ];

addParametersToModel[model_, par_] :=
  Block[{parameters, newParameters, newModel, unmodified},
   parameters = model[[4]];
   newParameters = Flatten[{par}][[ ;; , 1]];
   unmodified = Select[parameters, MemberQ[newParameters, #[[1]]] == False &];
   newParameters = Join[par, unmodified];
   newModel = model;
   newModel[[4]] = newParameters;
   newModel];

In[•]:= (*Model imported from JWS Online*)
modeldMF = ToExpression[ URLExecute["https://jjj.bio.vu.nl/models/li2/mf"]];
```

```
In[•]:= (*Local Sensitivity Analysis*)
model = modeldMF;
oldStSt = Quiet[ststSolMatFormR[model, 100 000]];
oldStStD = oldStSt;
vars = Select[oldStSt[[1]], #[[2]] > 10 &][[ ;; , 1]];
respTabD = Table[
   param = model[[4, j]];
   tempMod = addParametersToModel[model, {param[[1]] → param[[2]] * 1.00001}];
   newStStUp = Quiet[ststSolMatFormR[tempMod, 100 000]];
   tempMod = addParametersToModel[model, {param[[1]] → param[[2]] * 0.99999}];
   newStStDown = Quiet[ststSolMatFormR[tempMod, 100 000]];
   respTab = Table[{param[[1]], vars[[i]],
      ((vars[[i]] /. newStStUp[[1]]) - (vars[[i]] /. newStStDown[[1]])) /
       (2 * 0.00001 vars[[i]] /. oldStSt[[1]])}, {i, 1, Length[vars]}];
   respTab, {j, 1, Length[model[[4]]]}];
validPairsD = Select[Flatten[respTabD, 1], ! #[[3]] === 0. &][[ ;; , {1, 2}]];

In[•]:= (*Monte Carlo Random sampling and Robustness analysis*)
model = modeldMF;
pramD = modeldMF[[4]];
startingpoint = Table[#[[1]] → #[[2]] & /@ oldStStD[[i]], {i, 1, 2}];
pramnamesD = pramD[[ ;; , 1]];
pramvaluesD = pramD[[ ;; , 2]];
pramsizeD = Length[pramnamesD];
noOfParamsetsD = 30 000;
paramSetsMCd = ParallelTable[Table[
    pramnamesD[[i]] → RandomReal[{0.9 pramvaluesD[[i]], 1.1 pramvaluesD[[i]]}],
    {i, 1, Length[pramnamesD]}], {z, 1, noOfParamsetsD}];
ststD = ParallelTable[
  {Quiet[ststSolMatFormR[addParametersToModel[model, paramSetsMCd[[i]]], 100 000,
     startingpoint]], paramSetsMCd[[i]]}, {i, 1, Length[paramSetsMCd]}];
validStStD = Select[ststD, (model`M[t] /. #[[1, 1]]) > 1 &&
    (model`E[t] /. #[[1, 1]]) > 1.001 &];

In[•]:= validStStD = validStStD[[1 ;; 10 000]];
HvalsD = model`H[t] /. validStStD[[ ;; , 1, 1]];
MvalsD = model`M[t] /. validStStD[[ ;; , 1, 1]];
EvalsD = model`E[t] /. validStStD[[ ;; , 1, 1]];
IvalsD = model`I[t] /. validStStD[[ ;; , 1, 1]];
```

In[◦]:=
```
(*A box–and–whisker plot showing Robustness of iRBCs*)
IvalsDmed = IvalsD / Median[IvalsD];
bwplotY = BoxWhiskerChart[{IvalsDmed}, FrameLabel → {"", "iRBC"},
   ChartStyle → {Red}, LabelStyle → {FontSize → 16, FontFamily → "Helvetica"},
   FrameStyle → Thickness[0.003],
   BaseStyle → {FontSize → 20, FontFamily → "Helvetica"},
   ImageSize → 500, AspectRatio → 1 / 1.5]
```

Out[◦]=

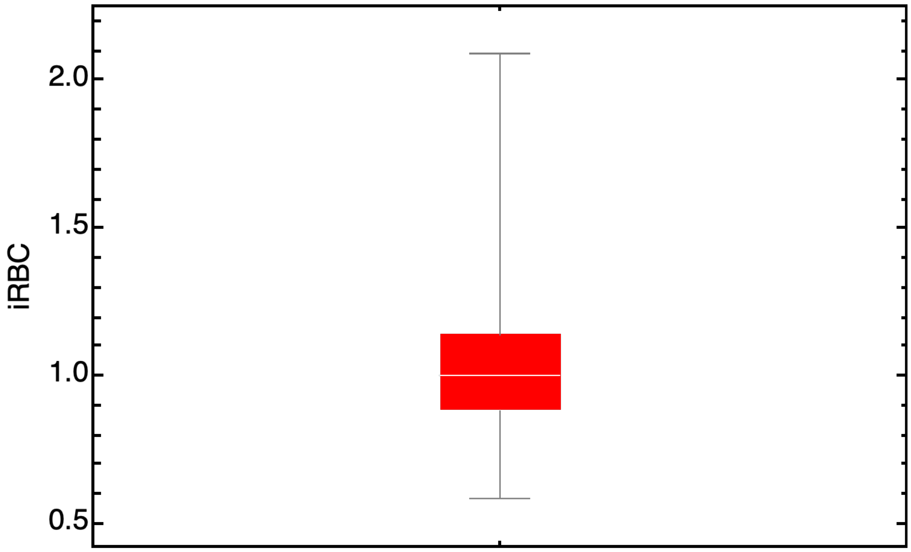

In[∘]:=
```
(*Robustness analysis of LSA*)
model = modeldMF;
validStSt = validStStD;
oldStSt = (*ststSolMatFormR[model,100000]*)oldStStD;
vars = Select[oldStSt[[1]], #[[2]] > 10 &][[ ;; , 1]];

parameterSpaceResponsesModelD = ParallelTable[
   paramSet = validStSt[[z, 2]];
   oldStSt = validStSt[[z, 1]];
   startingpoint = Table[#[[1]] → #[[2]] & /@ oldStSt[[i]], {i, 1, 2}];
   model = addParametersToModel[modeldMF, paramSet];
   paramSetResp = Table[
     param = paramSet[[j]];
     tempMod =
      addParametersToModel[model, {param[[1]] → param[[2]] * (*1.001*) 1.001}];
     newStStUp = Quiet[ststSolMatFormR[tempMod, 10 000, startingpoint]];
     tempMod =
      addParametersToModel[model, {param[[1]] → param[[2]] * (*0.999*) 0.999}];
     newStStDown = Quiet[ststSolMatFormR[tempMod, 10 000, startingpoint]];
     respTab = Table[
       If[(vars[[i]] /. newStStUp[[1]]) < 1 || (vars[[i]] /. newStStDown[[1]]) < 1,
        {param[[1]], vars[[i]], Null},
        {param[[1]], vars[[i]], ((vars[[i]] /. newStStUp[[1]]) -
            (vars[[i]] /. newStStDown[[1]])) /
          (2 * 0.001 vars[[i]] /. oldStSt[[1]]))}], {i, 1, Length[vars]}];
     respTab, {j, 1, Length[model[[4]]]}];
   {paramSetResp, paramSet},
   {z, 1, Length[validStSt]}];
parameterSpaceResponsesModelDFlat = Select[
   Flatten[parameterSpaceResponsesModelD[[ ;; , 1]], 2], ! #[[3]] === Null &];
```

In[∘]:=
```
validPairs = validPairsD;
respBigTabD = Table[
   paramName = model[[4, i, 1]];
   varName = vars[[j]];
   Select[parameterSpaceResponsesModelDFlat, #[[1]] === paramName &&
      #[[2]] === varName && MemberQ[validPairs, {paramName, varName}] == True &],
   {i, 1, Length[model[[4]]]}, {j, 1, Length[vars]}];
testTab = DeleteCases[Flatten[respBigTabD, 2], {}];
testTab2 = Partition[testTab, Length[validStStD]];
```

In[•]:=
```
(*Histograms *)
(*Note that alpha in the Li model is defined as beta in Article*)
tablaDli = Table[
  parName = StringReplace[ToString[testTab2[[i, 1, 1]]], "model`" → ""];
  varName = StringReplace[ToString[testTab2[[i, 1, 2]]], "model`" → ""];
  wtResponse = Select[Flatten[respTabD, 1],
     {#[[1]], #[[2]]} == {testTab2[[i, 1, 1]], testTab2[[i, 1, 2]]} &][[1, 3]];
  Show[Histogram[testTab2[[i, ;; , 3]],
    {Min[testTab2[[i, ;; , 3]]], Max[testTab2[[i, ;; , 3]]],
     Abs[Max[testTab2[[i, ;; , 3]]] - Min[testTab2[[i, ;; , 3]]]] / 50},
    "Probability", Frame → True, FrameLabel → {R_parName^varName, "Probablity"},
    LabelStyle → {FontSize → 16, FontFamily → "Helvetica"},
    FrameStyle → Thickness[0.003], PlotRange → {{0, 100}, {0, 0.25}},
    BaseStyle → {FontSize → 20, FontFamily → "Helvetica"}, ImageSize → 500,
    AspectRatio → 1 / 1.5, Axes → False, PlotRange → Automatic],
   ListLinePlot[{{wtResponse, 0}, {wtResponse, 1}},
    PlotStyle → Directive[Black, Opacity[0.7], Dashed, Thickness[0.005]]],
   PlotRange → Automatic],
  {i, 1, Length[testTab2]}];
(*tablaD[[1]]*)
```

In[•]:=
```
respTabD[[1, 3]]
```

Out[•]=
```
{model`alfa, model`I[t], 0.00841944}
```

In[•]:=
```
wtResponse = respTabD[[1, 3, 3]];
Show[Histogram[respBigTabD[[1, 3, ;; , 3]],
  {Min[respBigTabD[[1, 3, ;; , 3]]], Max[respBigTabD[[1, 3, ;; , 3]]],
   Abs[Max[respBigTabD[[1, 3, ;; , 3]]] - Min[respBigTabD[[1, 3, ;; , 3]]]] / 50},
  "Probability", Frame → True, FrameLabel → {R_alpha^iRBCs, "Probablity"},
  LabelStyle → {FontSize → 16, FontFamily → "Helvetica"},
  FrameStyle → Thickness[0.003], PlotRange → {{0, 100}, {0, 0.25}},
  BaseStyle → {FontSize → 20, FontFamily → "Helvetica"}, ImageSize → 500,
  AspectRatio → 1 / 1.5, Axes → False, PlotRange → {{0.004, 0.018}, {0, 0.07}}],
 ListLinePlot[{{wtResponse, 0}, {wtResponse, 1}},
  PlotStyle → Directive[Black, Opacity[0.7], Dashed, Thickness[0.005]]],
 PlotRange → {{0.004, 0.018}, {0, 0.07}}]
```

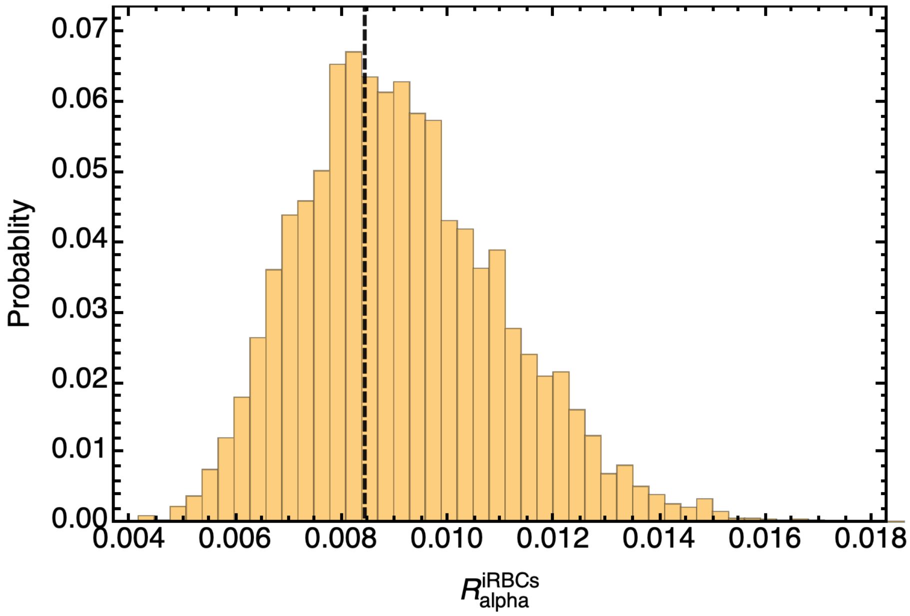

In[∘]:=
```
Export["aa.pdf", aa, ImageResolution → 600];
```

In[∘]:=
```
respTabD[[1, 1]]
```

Out[∘]=
```
{model`alfa, model`E[t], 78.2529}
```

In[∘]:=
```
respBigTabD[[1, 1, 1]]
```

Out[∘]=
```
{model`alfa, model`E[t], 7.07879}
```

In[∘]:=
```
wtResponse = respTabD[[1, 1, 3]];
Show[Histogram[respBigTabD[[1, 1, ;; , 3]],
  {Min[respBigTabD[[1, 1, ;; , 3]]], Max[respBigTabD[[1, 1, ;; , 3]]],
   Abs[Max[respBigTabD[[1, 1, ;; , 3]]] - Min[respBigTabD[[1, 1, ;; , 3]]]] / 400},
  "Probability", Frame → True, FrameLabel → {R^(Immune Cells)_alpha, "Probablity"},
  LabelStyle → {FontSize → 16, FontFamily → "Helvetica"},
  FrameStyle → Thickness[0.003], PlotRange → {{0, 100}, {0, 0.25}},
  BaseStyle → {FontSize → 20, FontFamily → "Helvetica"}, ImageSize → 500,
  AspectRatio → 1 / 1.5, Axes → False, PlotRange → {{0, 100}, {0, 0.25}}],
 ListLinePlot[{{wtResponse, 0}, {wtResponse, 1}},
  PlotStyle → Directive[Black, Opacity[0.7], Dashed, Thickness[0.005]]],
 PlotRange → {{0, 100}, {0, 0.25}}]
```

Out[∘]=

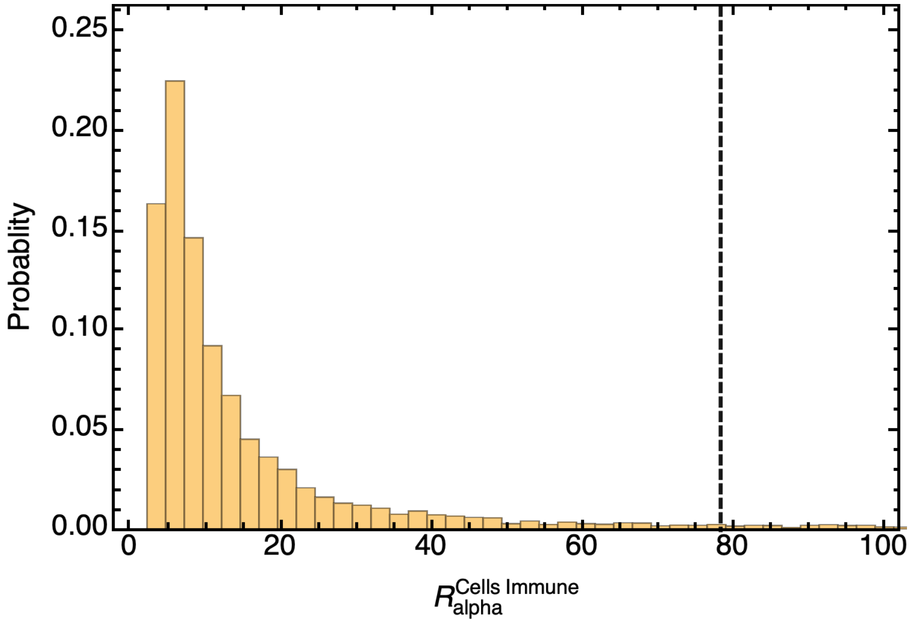

Uncertainty Analysis

```
In[]:= model = modeldMF;
oldStSt = ststSolMatFormR[model, 100 000];
vars = Select[oldStSt[[1]], #[[2]] > 10 &][[ ;; , 1]];
oldStStD = oldStSt;
startingpoint = Table[#[[1]] → #[[2]] & /@ oldStStD[[i]], {i, 1, 2}];
paramsToScan = model[[4]];
paramNames = paramsToScan[[ ;; , 1]];
paramVals = paramsToScan[[ ;; , 2]];
paramLogExtremes = Table[
   var = 0.10;
   paramNames[[i]] → {(1 - var) * paramVals[[i]], (1 + var) * paramVals[[i]]},
   {i, 1, Length[paramNames]}];

paramLogVariance =
  #[[1]] → If[#[[2]] ≠ {0., 0.}, Variance[Log[RandomVariate[UniformDistribution[
          {#[[2, 1]], #[[2, 2]]}], 10 000 000]]], 0] & /@ paramLogExtremes;

respTabD = Flatten[Table[
    param = model[[4, j]];
    tempMod = addParametersToModel[model, {param[[1]] → param[[2]] * 1.0001}];
    newStStUp = Quiet[ststSolMatFormR[tempMod, 100 000, startingpoint]];
    respTab = Table[{param[[1]], vars[[i]], (param[[1]] /. paramLogVariance) *
         (((vars[[i]] /. newStStUp[[1]]) - (vars[[i]] /. oldStSt[[1]])) /
            (0.0001)) ^ 2}, {i, 1, Length[vars]}];
    respTab, {j, 1, Length[model[[4]]]}], 1];

overallVariances =
  Thread[vars → (Total[#] & /@ Table[Select[respTabD, #[[2]] == vars[[i]] &&
            ! #[[3]] === Indeterminate &], {i, 1, Length[vars]}][[ ;; , ;; , 3]])];

contributionOfPars = Table[
   num = Flatten[
      Select[respTabD, #[[1]] == paramNames[[i]] && #[[2]] == vars[[j]] &]][[3]];
   denom = vars[[j]] /. overallVariances;
   {paramNames[[i]], vars[[j]], num / denom * 100},
   {j, 1, Length[vars]}, {i, 1, Length[paramNames]}];
TableForm[contributionOfPars]
```

Out[∘]//TableForm=

model`alfa<br>model`E[t]<br>14.896	model`beta<br>model`E[t]<br>0.342047	model`d1<br>model`E[t]<br>14.8971	model`d2<br>model`E[t]<br>1.18057	model`delta<br>model`E[t]<br>18.0161	model`gam<br>model`E[t<br>0.0047793
model`alfa<br>model`H[t]<br>0.484009	model`beta<br>model`H[t]<br>1.2188	model`d1<br>model`H[t]<br>40.7712	model`d2<br>model`H[t]<br>3.78104	model`delta<br>model`H[t]<br>0.000378568	model`gam<br>model`H[t<br>0.0156402
model`alfa<br>model`I[t]<br>0.000462612	model`beta<br>model`I[t]<br>15.5221	model`d1<br>model`I[t]<br>0.000462645	model`d2<br>model`I[t]<br>48.1376	model`delta<br>model`I[t]<br>0.000559509	model`gam<br>model`I[t<br>0.198731
model`alfa<br>model`M[t]<br>0.00376044	model`beta<br>model`M[t]<br>14.6431	model`d1<br>model`M[t]<br>0.00376074	model`d2<br>model`M[t]<br>45.428	model`delta<br>model`M[t]<br>0.00454813	model`gam<br>model`M[t<br>0.187903

In[∘]:=
```
NotebookSave[];
```

Additional file 4 : "Uncovering the effects of heterogeneity and parameter sensitivity on within-host dynamics of disease: malaria as a case study"

In[◦]:=

Niger Analysis

In[•]:=
```
(*Helper functions defined for steady state
  determination and parameter modifications*)
ststSolMatFormR[model_, time_ : 100, startingpoint_ : {}] :=
  Block[{odes, rateEquations, assignments, parameters,
    initialValues, variables, timeCourse, findRootEquations,
    findRootVariables, steadyStateVariables, fluxes},
   odes = Thread[D[model[[1, 1]], t] == model[[1, 2]].model[[1, 3]]];
   rateEquations = model[[2]];
   assignments = model[[5]];
   parameters = model[[4]];
   initialValues = model[[3]];
   variables = model[[1, 1]];

   (*Steady-state solution initialized with result of time evolution*)
   findRootEquations = odes /. D[_[t], t] → 0;
findRootVariables = If[startingpoint == {},
     timeCourse = NDSolve[Join[odes, initialValues] //. rateEquations //.
         assignments //. parameters, variables, {t, 0, time},
       (*Method→{"StiffnessSwitching","NonstiffTest"→"NormBound"}*)
       MaxSteps → Infinity, Method → "BDF", AccuracyGoal → 10,
       PrecisionGoal → 10, MaxStepSize → time / 1000.];
     Partition[Flatten[{#, # /. timeCourse /. t → time} & /@ variables], 2],
     Partition[Flatten[{#[[1]], #[[2]]} & /@ startingpoint[[1]]], 2]];
steadyStateVariables = FindRoot[findRootEquations //. rateEquations //.
       assignments //. parameters, findRootVariables, MaxIterations → 1000];
fluxes = # //. assignments //. parameters /. steadyStateVariables & /@
     rateEquations;
   {steadyStateVariables, fluxes}
  ];

addParametersToModel[model_, par_] :=
  Block[{parameters, newParameters, newModel, unmodified},
   parameters = model[[4]];
   newParameters = Flatten[{par}][[ ;; , 1]];
   unmodified = Select[parameters, MemberQ[newParameters, #[[1]]] == False &];
   newParameters = Join[par, unmodified];
   newModel = model;
   newModel[[4]] = newParameters;
   newModel];
```

In[•]:=
```
(*Model imported from JWS Online*)
modelbMF = ToExpression[ URLExecute["https://jjj.bio.vu.nl/models/niger1/mf"]];
```

```
In[•]:= (*Local Sensitivity Analysis*)
model = modelbMF;
(*reference steady state*)
oldStSt = Quiet[ststSolMatFormR[model, 100 000]];
oldStStB = oldStSt;
vars1 = Join[{Select[oldStSt[[1]], #[[2]] > 10 &][[ ;; , 1]]},
   {model`Yt /. modelbMF[[5]]}];
vars = {vars1[[1, 1]], vars1[[1, 2]], vars1[[1, 3]], vars1[[1, 4]], vars1[[1, 5]],
   vars1[[1, 6]], vars1[[1, 7]], vars1[[1, 8]], vars1[[1, 9]], vars1[[2]]};
respTabB = Table[
   param = model[[4, j]];
   (*steady state with up perturbation*)
   tempMod = addParametersToModel[model, {param[[1]] → param[[2]] * 1.001}];
   newStStUp = Quiet[ststSolMatFormR[tempMod, 100 000]];
   (*steady state with down perturbation*)
   tempMod = addParametersToModel[model, {param[[1]] → param[[2]] * 0.999}];
   newStStDown = Quiet[ststSolMatFormR[tempMod, 100 000]];
   (*response coefficient calculated*)
   respTab = Table[{param[[1]], vars[[i]],
      ((vars[[i]] /. newStStUp[[1]]) - (vars[[i]] /. newStStDown[[1]])) /
       (2 * 0.001 vars[[i]] /. oldStSt[[1]])}, {i, 1, Length[vars]}];
   respTab, {j, 1, Length[model[[4]]]}];
validPairsB = Select[Flatten[respTabB, 1], ! #[[3]] === 0. &][[ ;; , {1, 2}]];
respTabB[[ ;; , 10, 2]] = "model`Yt";
```

In[◦]:=
```
(*Monte Carlo Random sampling and Robustness analysis*)
model = modelbMF;
pramB = modelbMF[[4]];
startingpoint = Table[#[[1]] → #[[2]] & /@ oldStStB[[i]], {i, 1, 2}];
pramnamesB = pramB[[ ;; , 1]];
pramvaluesB = pramB[[ ;; , 2]];
pramsizeB = Length[pramnamesB];
noOfParamsetsB = 10 000;
paramSetsMC = ParallelTable[Table[
    pramnamesB[[i]] → RandomReal[{0.9 pramvaluesB[[i]], 1.1 pramvaluesB[[i]]}],
    {i, 1, Length[pramnamesB]}], {z, 1, noOfParamsetsB}];
paramSetsMCb = paramSetsMC;
ststB = ParallelTable[
   {Quiet[ststSolMatFormR[
      addParametersToModel[model, paramSetsMCb[[i]]], 100 000, startingpoint]],
    paramSetsMCb[[i]]}, {i, 1, Length[paramSetsMCb]}];
validStStB = ststB;
XvalsB = model`X[t] /. validStStB[[ ;; , 1, 1]];
MvalsB = model`M[t] /. validStStB[[ ;; , 1, 1]];
YtvalsB = model`Yt /. model[[5]] /. validStStB[[ ;; , 1, 1]];
BvalsB = model`B[t] /. validStStB[[ ;; , 1, 1]];
AvalsB = model`A[t] /. validStStB[[ ;; , 1, 1]];
```

In[◦]:=
```
(*To Export each variable's achieved steady states for each parameter set*)
(*Export[NotebookDirectory[]<>"NigerXss.txt",XvalsB];
Export[NotebookDirectory[]<>"NigerMss.txt",MvalsB];
Export[NotebookDirectory[]<>"NigerYtss.txt",YtvalsB];
Export[NotebookDirectory[]<>"NigerBss.txt",BvalsB];
Export[NotebookDirectory[]<>"NigerAss.txt",AvalsB];*)
```

In[∘]:=
```
(*A box–and–whisker plot showing Robustness of iRBCs*)
YtvalsBmed = YtvalsB / Median[YtvalsB];
bwplotY = BoxWhiskerChart[{YtvalsBmed}, FrameLabel → {"", "iRBC"},
  ChartStyle → {Red}, LabelStyle → {FontSize → 16, FontFamily → "Helvetica"},
  FrameStyle → Thickness[0.003],
  BaseStyle → {FontSize → 20, FontFamily → "Helvetica"},
  ImageSize → 500, AspectRatio → 1 / 1.5]
```

Out[∘]=

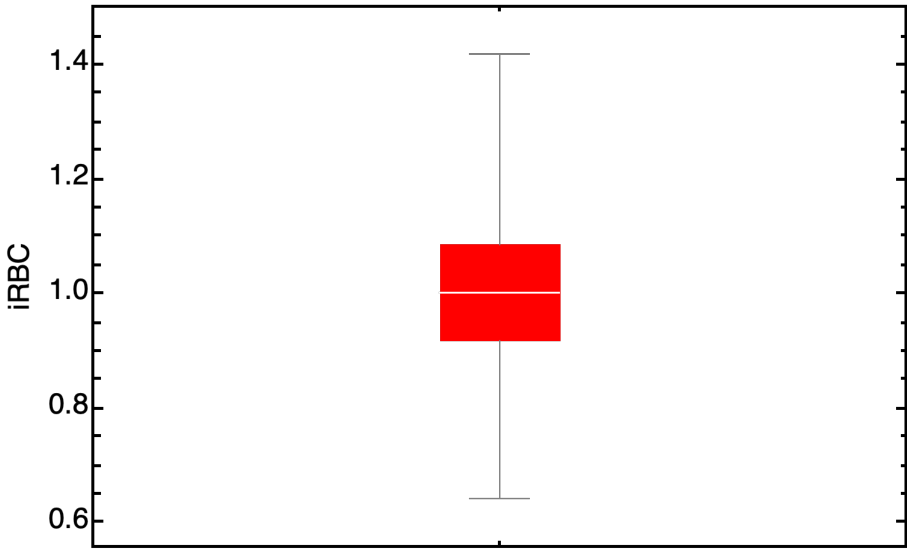

```
In[•]:= (*Robustness analysis of LSA*)
model = modelbMF;
validStSt = validStStB;
oldStSt = oldStStB;
vars1 = Join[{Select[oldStSt[[1]], #[[2]] > 10 &][[ ;; , 1]]},
   {model`Yt /. modelbMF[[5]]}];
vars = {vars1[[1, 1]], vars1[[1, 2]], vars1[[1, 3]], vars1[[1, 4]], vars1[[1, 5]],
   vars1[[1, 6]], vars1[[1, 7]], vars1[[1, 8]], vars1[[1, 9]], vars1[[2]]};
parameterSpaceResponsesModelB = ParallelTable[
   paramSet = validStSt[[z, 2]];
   oldStSt = validStSt[[z, 1]];
   startingpoint = Table[#[[1]] → #[[2]] & /@ oldStSt[[i]], {i, 1, 2}];
   model = addParametersToModel[modelbMF, paramSet];
   paramSetResp = Table[
     param = paramSet[[j]];
     tempMod =
      addParametersToModel[model, {param[[1]] → param[[2]] * (*1.001*) 1.001}];
     newStStUp = Quiet[ststSolMatFormR[tempMod, 10 000, startingpoint]];
     tempMod =
      addParametersToModel[model, {param[[1]] → param[[2]] * (*0.999*) 0.999}];
     newStStDown = Quiet[ststSolMatFormR[tempMod, 10 000, startingpoint]];
     respTab = Table[{param[[1]], vars[[i]],
        ((vars[[i]] /. newStStUp[[1]]) - (vars[[i]] /. newStStDown[[1]])) /
         (2 * 0.001 vars[[i]] /. oldStSt[[1]])}, {i, 1, Length[vars]}];
     respTab, {j, 1, Length[model[[4]]]}];
   {paramSetResp, paramSet},
   {z, 1, Length[validStSt]}];
parameterSpaceResponsesModelBFlat = Select[
   Flatten[parameterSpaceResponsesModelB[[ ;; , 1]], 2], ! #[[3]] === Null &];

In[•]:= validPairs = validPairsB;
respBigTabB = Table[
   paramName = model[[4, i, 1]];
   varName = vars[[j]];
   Select[parameterSpaceResponsesModelBFlat,
    #[[1]] == paramName && #[[2]] == varName &],
   {i, 1, Length[model[[4]]]}, {j, 1, Length[vars]}];
testTab = DeleteCases[Flatten[respBigTabB, 2], {}];
testTab2 = Partition[testTab, Length[validStStB]];
testTab2[[10, ;; , 2]] = "model`Yt";

In[•]:= respTabB[[1, 10]]

Out[•]= {model`beta, model`Yt, 0.0761974}
```

In[*]:=
```
(*Histograms*)
tablaBniger = Table[
  parName = StringReplace[ToString[testTab2[[i, 1, 1]]], "model`" → ""];
  varName = StringReplace[ToString[testTab2[[i, 1, 2]]], "model`" → ""];
  wtResponse = Select[Flatten[respTabB, 1],
     {#[[1]], #[[2]]} == {testTab2[[i, 1, 1]], testTab2[[i, 1, 2]]} &][[1, 3]];
  Show[Histogram[testTab2[[i, ;; , 3]], {Min[testTab2[[i, ;; , 3]]],
     Max[testTab2[[i, ;; , 3]]] + 0.0001,
     Abs[Max[testTab2[[i, ;; , 3]]] - Min[testTab2[[i, ;; , 3]]]] / 50},
    Probability, Frame → True, FrameLabel → {R^varName_parName, "Probablity"},
    LabelStyle → {FontSize → 16, FontFamily → "Helvetica"},
    FrameStyle → Thickness[0.003], PlotRange → {{0.02, 0.2}, {0, 0.08}},
    BaseStyle → {FontSize → 20, FontFamily → "Helvetica"}, ImageSize → 500,
    AspectRatio → 1 / 1, Axes → False, PlotRange → {All, All} ],
   ListLinePlot[{{wtResponse, 0}, {wtResponse, 1}},
    PlotStyle → Directive[Black, Opacity[0.7], Dashed, Thickness[0.005]]],
   PlotRange → {{0.02, 0.2}, {0, 0.08}}],
  {i, 10, 10(*Length[testTab2]*)}]
```

Out[*]= { 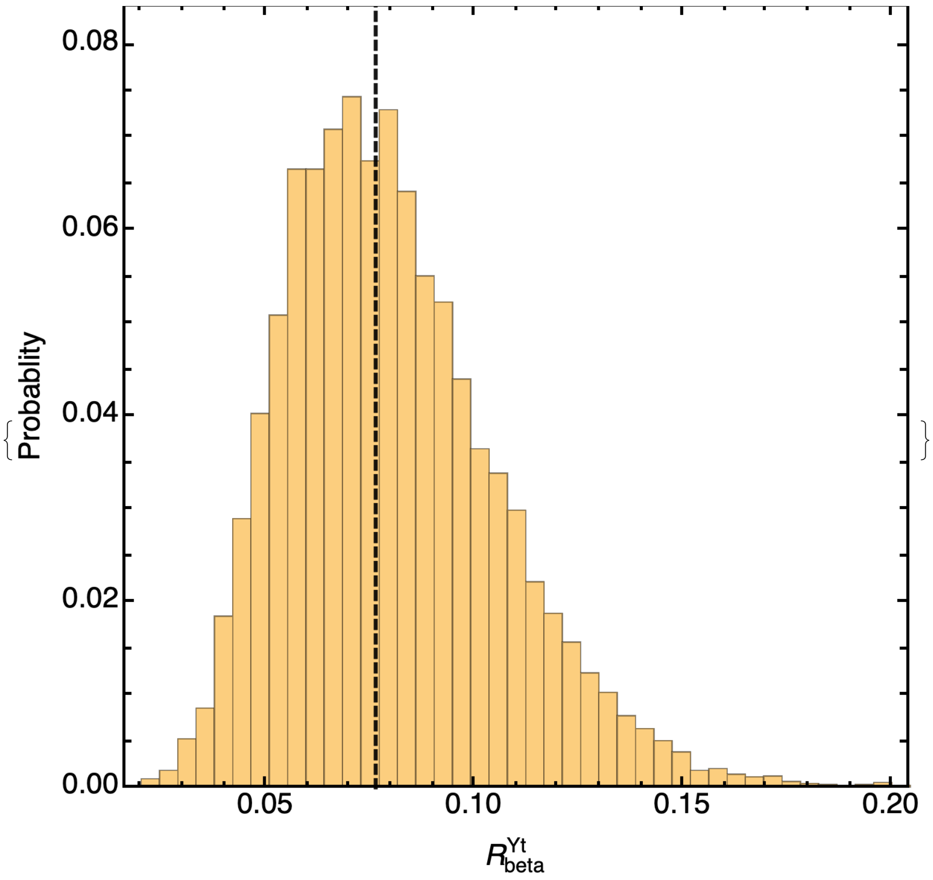 }

In[*]:=
```
(*tablaBniger*)
```

Uncertainty analysis

In[•]:=
```
model = modelbMF;
(*wildtype steady state*)
oldStSt = Quiet[ststSolMatFormR[model, 100 000]];
vars1 = Join[{Select[oldStSt[[1]], #[[2]] > 10 &][[ ;; , 1]]},
   {model`Yt /. modelbMF[[5]]}];
vars = {vars1[[1, 1]], vars1[[1, 2]], vars1[[1, 3]], vars1[[1, 4]], vars1[[1, 5]],
   vars1[[1, 6]], vars1[[1, 7]], vars1[[1, 8]], vars1[[1, 9]], vars1[[2]]};
oldStStB = oldStSt;
startingpoint = Table[#[[1]] → #[[2]] & /@ oldStStB[[i]], {i, 1, 2}];
paramsToScan = model[[4]];
paramNames = paramsToScan[[ ;; , 1]];
paramVals = paramsToScan[[ ;; , 2]];
paramLogExtremes = Table[
   var = 0.10;
   paramNames[[i]] → {(1 - var) * paramVals[[i]], (1 + var) * paramVals[[i]]},
   {i, 1, Length[paramNames]}];

paramLogVariance =
  #[[1]] → If[#[[2]] ≠ {0., 0.}, Variance[Log[RandomVariate[UniformDistribution[
          {#[[2, 1]], #[[2, 2]]}], 10 000 000]]], 0] & /@ paramLogExtremes;

respTabB = Flatten[Table[
    param = model[[4, j]];
    tempMod = addParametersToModel[model, {param[[1]] → param[[2]] * 1.0001}];
    newStStUp = Quiet[ststSolMatFormR[tempMod, 100 000, startingpoint]];

    respTab = Table[{param[[1]], vars[[i]], (param[[1]] /. paramLogVariance) *
        (((vars[[i]] /. newStStUp[[1]]) - (vars[[i]] /. oldStSt[[1]])) /
          (0.0001)) ^ 2}, {i, 1, Length[vars]}];
    respTab, {j, 1, Length[model[[4]]]}], 1];

overallVariances =
  Thread[vars → (Total[#] & /@ Table[Select[respTabB, #[[2]] == vars[[i]] &&
          ! #[[3]] === Indeterminate &], {i, 1, Length[vars]}][[ ;; , ;; , 3]])];

contributionOfPars = Table[
   num = Flatten[
      Select[respTabB, #[[1]] == paramNames[[i]] && #[[2]] == vars[[j]] &]][[3]];
   denom = vars[[j]] /. overallVariances;
   {paramNames[[i]], vars[[j]], num / denom * 100},
   {j, 1, Length[vars]}, {i, 1, Length[paramNames]}];
contributionOfPars[[10, ;; , 2]] = "model`Yt[t]";
TableForm[contributionOfPars]
```

Out[∘]//TableForm=

model\`beta<br>model\`A[t]<br>2.54706	model\`eta<br>model\`A[t]<br>8.24446	model\`gamma1<br>model\`A[t]<br>10.6433	model\`gamma2<br>model\`A[t]<br>2.4487	model\`gamma3<br>model\`A[t]<br>2.44852	mo[illegible]<br>mo[illegible]<br>2.[illegible]
model\`beta<br>model\`B[t]<br>3.87088	model\`eta<br>model\`B[t]<br>9.93337×10^{-23}	model\`gamma1<br>model\`B[t]<br>5.48087	model\`gamma2<br>model\`B[t]<br>5.83039	model\`gamma3<br>model\`B[t]<br>5.82995	mo[illegible]<br>mo[illegible]<br>5.[illegible]
model\`beta<br>model\`M[t]<br>8.57714	model\`eta<br>model\`M[t]<br>0.	model\`gamma1<br>model\`M[t]<br>9.4767	model\`gamma2<br>model\`M[t]<br>0.785341	model\`gamma3<br>model\`M[t]<br>0.785281	mo[illegible]<br>mo[illegible]<br>0.[illegible]
model\`beta<br>model\`X[t]<br>0.0069387	model\`eta<br>model\`X[t]<br>0.	model\`gamma1<br>model\`X[t]<br>0.126059	model\`gamma2<br>model\`X[t]<br>0.0104467	model\`gamma3<br>model\`X[t]<br>0.0104459	mo[illegible]<br>mo[illegible]<br>0.[illegible]
model\`beta<br>model\`Y1[t]<br>0.4352	model\`eta<br>model\`Y1[t]<br>3.33425×10^{-23}	model\`gamma1<br>model\`Y1[t]<br>0.516157	model\`gamma2<br>model\`Y1[t]<br>0.655104	model\`gamma3<br>model\`Y1[t]<br>0.655054	mo[illegible]<br>mo[illegible]<br>0.[illegible]
model\`beta<br>model\`Y2[t]<br>0.112709	model\`eta<br>model\`Y2[t]<br>7.43671×10^{-23}	model\`gamma1<br>model\`Y2[t]<br>22.3536	model\`gamma2<br>model\`Y2[t]<br>4.6191	model\`gamma3<br>model\`Y2[t]<br>0.169572	mo[illegible]<br>mo[illegible]<br>0.[illegible]
model\`beta<br>model\`Y3[t]<br>0.0090932	model\`eta<br>model\`Y3[t]<br>0.	model\`gamma1<br>model\`Y3[t]<br>19.3576	model\`gamma2<br>model\`Y3[t]<br>3.70578	model\`gamma3<br>model\`Y3[t]<br>5.85219	mo[illegible]<br>mo[illegible]<br>0.[illegible]
model\`beta<br>model\`Y4[t]<br>0.019754	model\`eta<br>model\`Y4[t]<br>3.85929×10^{-23}	model\`gamma1<br>model\`Y4[t]<br>16.6728	model\`gamma2<br>model\`Y4[t]<br>2.62577	model\`gamma3<br>model\`Y4[t]<br>2.62557	mo[illegible]<br>mo[illegible]<br>7.[illegible]
model\`beta<br>model\`Y5[t]<br>0.114075	model\`eta<br>model\`Y5[t]<br>4.04947×10^{-23}	model\`gamma1<br>model\`Y5[t]<br>14.8491	model\`gamma2<br>model\`Y5[t]<br>1.90565	model\`gamma3<br>model\`Y5[t]<br>1.90551	mo[illegible]<br>mo[illegible]<br>1.[illegible]
model\`beta<br>model\`Yt[t]<br>0.140592	model\`eta<br>model\`Yt[t]<br>1.03527×10^{-22}	model\`gamma1<br>model\`Yt[t]<br>9.52759	model\`gamma2<br>model\`Yt[t]<br>0.0853789	model\`gamma3<br>model\`Yt[t]<br>0.0853723	mo[illegible]<br>mo[illegible]<br>0.[illegible]

In[∘]:=

```
NotebookSave[];
```

Additional file 5 : "Uncovering the effects of heterogeneity and parameter sensitivity on within-host dynamics of disease: malaria as a case study"

In[∘]:=

Okrinya Analysis

In[•]:=
```
(*Helper functions defined for steady state
  determination and parameter modifications*)
ststSolMatFormR[model_, time_ : 100, startingpoint_ : {}] :=
  Block[{odes, rateEquations, assignments, parameters,
    initialValues, variables, timeCourse, findRootEquations,
    findRootVariables, steadyStateVariables, fluxes},
   odes = Thread[D[model[[1, 1]], t] == model[[1, 2]].model[[1, 3]]];
   rateEquations = model[[2]];
   assignments = model[[5]];
   parameters = model[[4]];
   initialValues = model[[3]];
   variables = model[[1, 1]];

   (*Steady-state solution initialized with result of time evolution*)
   findRootEquations = odes /. D[_[t], t] → 0;
findRootVariables = If[startingpoint == {},
     timeCourse = NDSolve[Join[odes, initialValues] //. rateEquations //.
         assignments //. parameters, variables, {t, 0, time},
       (*Method→{"StiffnessSwitching","NonstiffTest"→"NormBound"}*)
       MaxSteps → Infinity, Method → "BDF", AccuracyGoal → 10,
       PrecisionGoal → 10, MaxStepSize → time / 1000.];
     Partition[Flatten[{#, # /. timeCourse /. t → time} & /@ variables], 2],
     Partition[Flatten[{#[[1]], #[[2]]} & /@ startingpoint[[1]]], 2]];
steadyStateVariables = FindRoot[findRootEquations //. rateEquations //.
       assignments //. parameters, findRootVariables, MaxIterations → 1000];
fluxes = # //. assignments //. parameters /. steadyStateVariables & /@
     rateEquations;
   {steadyStateVariables, fluxes}
  ];

addParametersToModel[model_, par_] :=
  Block[{parameters, newParameters, newModel, unmodified},
   parameters = model[[4]];
   newParameters = Flatten[{par}][[ ;; , 1]];
   unmodified = Select[parameters, MemberQ[newParameters, #[[1]]] == False &];
   newParameters = Join[par, unmodified];
   newModel = model;
   newModel[[4]] = newParameters;
   newModel];
```

In[•]:=
```
(*Model imported from JWS Online*)
modelaMF =
  ToExpression[ URLExecute["https://jjj.bio.vu.nl/models/okrinya1/mf"]];
```

In[•]:=
```
(*Local Sensitivity Analysis*)
model = modelaMF;
oldStSt = Quiet[ststSolMatFormR[model, 100 000]];
oldStStA = oldStSt;
startingpoint = Table[#[[1]] → #[[2]] & /@ oldStSt[[i]], {i, 1, 2}];
vars = Select[oldStSt[[1]], #[[2]] > 0.001 &][[ ;; , 1]];
respTabA = Table[
   param = model[[4, j]];
   tempMod = addParametersToModel[model, {param[[1]] → param[[2]] * 1.001}];
   newStStUp = Quiet[ststSolMatFormR[tempMod, 100 000, startingpoint]];
   tempMod = addParametersToModel[model, {param[[1]] → param[[2]] * 0.999}];
   newStStDown = Quiet[ststSolMatFormR[tempMod, 100 000, startingpoint]];
   respTab = Table[{param[[1]], vars[[i]],
      ((vars[[i]] /. newStStUp[[1]]) - (vars[[i]] /. newStStDown[[1]])) /
       (2 * 0.001 vars[[i]] /. oldStSt[[1]])}, {i, 1, Length[vars]}];
   respTab, {j, 1, Length[model[[4]]]}];
validPairsA = Select[Flatten[respTabA, 1], ! #[[3]] === 0. &][[ ;; , {1, 2}]];
```

In[•]:=
```
(*Monte Carlo Random sampling and Robustness analysis*)
model = modelaMF;
pramA = modelaMF[[4]];
startingpoint = Table[#[[1]] → #[[2]] & /@ oldStStA[[i]], {i, 1, 2}];
pramnamesA = pramA[[ ;; , 1]];
pramvaluesA = pramA[[ ;; , 2]];
pramsizeA = Length[pramnamesA];
noOfParamsetsA = 10 000;
paramSetsMC = ParallelTable[Table[
    pramnamesA[[i]] → RandomReal[{0.9 pramvaluesA[[i]], 1.1 pramvaluesA[[i]]}],
    {i, 1, Length[pramnamesA]}], {z, 1, noOfParamsetsA}];
paramSetsMCa = paramSetsMC;
ststA = ParallelTable[
   {Quiet[ststSolMatFormR[addParametersToModel[model, paramSetsMCa[[i]]], 10,
      startingpoint]], paramSetsMCa[[i]]}, {i, 1, Length[paramSetsMCa]}];
validStStA = Select[ststA, (model`X[t] /. #[[1, 1]]) > 0.001 &&
     (model`Y[t] /. #[[1, 1]]) > 0.001 &];
XvalsA = model`X[t] /. validStStA[[ ;; , 1, 1]];
YvalsA = model`Y[t] /. validStStA[[ ;; , 1, 1]];
MvalsA = model`M[t] /. validStStA[[ ;; , 1, 1]];
GvalsA = model`G[t] /. validStStA[[ ;; , 1, 1]];
PvalsA = model`P[t] /. validStStA[[ ;; , 1, 1]];
AvalsA = model`A[t] /. validStStA[[ ;; , 1, 1]];
```

In[∘]:=
```
(*A box–and–whisker plot showing Robustness of iRBCs*)
YvalsAmed = YvalsA / Median[YvalsA];
bwplotY = BoxWhiskerChart[{YvalsAmed}, FrameLabel → {"", "iRBC  (cells /μL)"},
  ChartStyle → {Red}, LabelStyle → {FontSize → 16, FontFamily → "Helvetica"},
  FrameStyle → Thickness[0.003],
  BaseStyle → {FontSize → 20, FontFamily → "Helvetica"},
  ImageSize → 500, AspectRatio → 1 / 1.5]
```

Out[∘]=

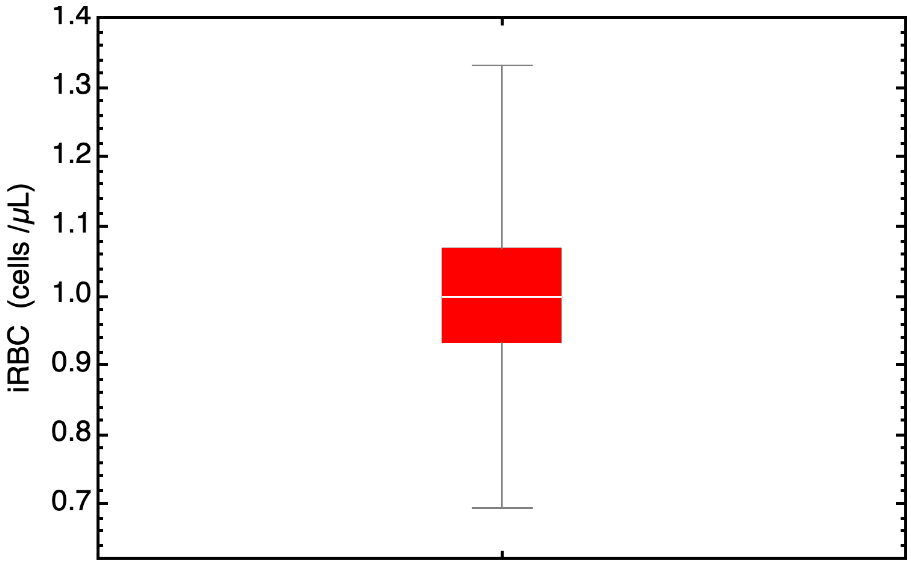

```
In[]:= (*Robustness analysis of LSA*)
model = modelaMF;
validStSt = validStStA;
oldStSt = (*ststSolMatFormR[model,100000]*)oldStStA;
vars = Select[oldStSt[[1]], #[[2]] > 0.0001 &][[ ;; , 1]];
parameterSpaceResponsesModelA = ParallelTable[
   paramSet = validStSt[[z, 2]];
   oldStSt = validStSt[[z, 1]];
   startingpoint = Table[#[[1]] → #[[2]] & /@ oldStSt[[i]], {i, 1, 2}];
   model = addParametersToModel[modelaMF, paramSet];
   paramSetResp = Table[
     param = paramSet[[j]];
     tempMod = addParametersToModel[model,
       {param[[1]] → param[[2]] * 1.001}];
     newStStUp = Quiet[ststSolMatFormR[tempMod, 10 000, startingpoint]];
     tempMod = addParametersToModel[model,
       {param[[1]] → param[[2]] * 0.999}];
     newStStDown = Quiet[ststSolMatFormR[tempMod, 10 000, startingpoint]];
     respTab = Table[
       {param[[1]], vars[[i]],
        ((vars[[i]] /. newStStUp[[1]]) - (vars[[i]] /. newStStDown[[1]])) /
         (2 * 0.001 vars[[i]] /. oldStSt[[1]])}, {i, 1, Length[vars]}];
     respTab, {j, 1, Length[model[[4]]]}];
   {paramSetResp, paramSet},
   {z, 1, Length[validStSt]}];
parameterSpaceResponsesModelAFlat = Select[
   Flatten[parameterSpaceResponsesModelA[[ ;; , 1]], 2], ! #[[3]] === Null &];
validPairs = validPairsA;
respBigTabA = Table[
   paramName = model[[4, i, 1]];
   varName = vars[[j]];
   Select[parameterSpaceResponsesModelAFlat, #[[1]] === paramName &&
      #[[2]] === varName && MemberQ[validPairs, {paramName, varName}] == True &],
   {i, 1, Length[model[[4]]]}, {j, 1, Length[vars]}];

In[]:= testTab = DeleteCases[Flatten[respBigTabA, 2], {}];
testTab2 = Partition[testTab, Length[validStStA]];

In[]:= testTab2[[12, 1]]

Out[]= {model`betaX, model`Y[t], 0.354907}
```

In[∘]:=

```
(*Histograms*)
tablaAokrinya = Table[
  parName = StringReplace[ToString[testTab2[[i, 1, 1]]], "model`" → ""];
  varName = StringReplace[ToString[testTab2[[i, 1, 2]]], "model`" → ""];
  wtResponse = Select[Flatten[respTabA, 1],
     {#[[1]], #[[2]]} == {testTab2[[i, 1, 1]], testTab2[[i, 1, 2]]} &][[1, 3]];
  Show[Histogram[testTab2[[i, ;; , 3]],
    {Min[testTab2[[i, ;; , 3]]], Max[testTab2[[i, ;; , 3]]],
     Abs[Max[testTab2[[i, ;; , 3]]] - Min[testTab2[[i, ;; , 3]]]] / 50},
    Probability, Frame → True, FrameLabel → {R^varName_parName, "Probablity"},
    LabelStyle → {FontSize → 16, FontFamily → "Helvetica"},
    FrameStyle → Thickness[0.003], PlotRange → All,
    BaseStyle → {FontSize → 20, FontFamily → "Helvetica"}, ImageSize → 500,
    AspectRatio → 1 / 1.5, Axes → False, PlotRange → Automatic ],
   ListLinePlot[{{wtResponse, 0}, {wtResponse, 1}},
    PlotStyle → Directive[Black, Opacity[0.7], Dashed, Thickness[0.005]]],
   PlotRange → {{0.2, 0.8}, {0, 0.08}}],
  {i, 12, (*Length[testTab2]*)12}]
```

Out[∘]= {

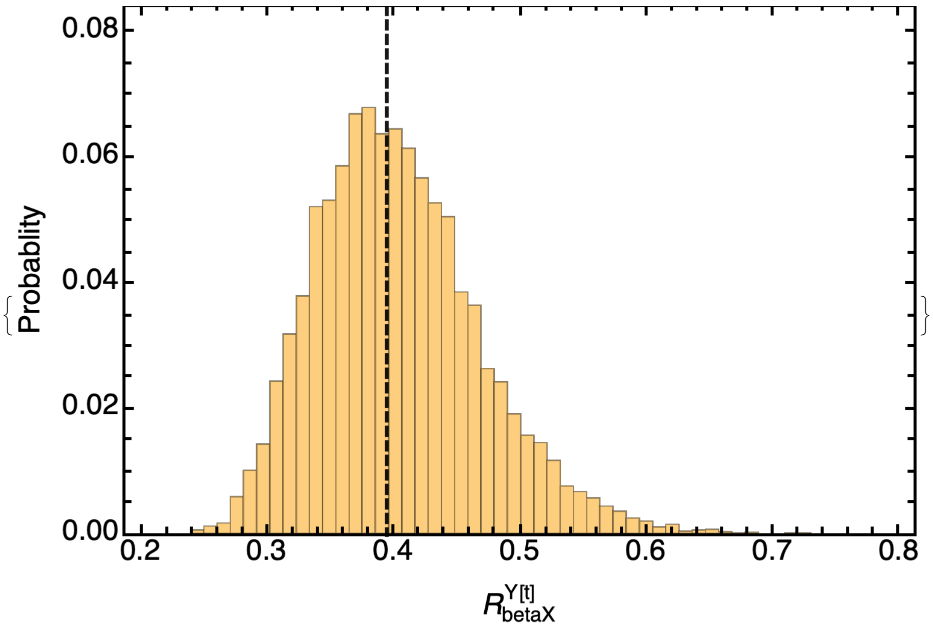

}

Uncertainty analysis

```
In[•]:= model = modelaMF;
oldStSt = oldStStA;
vars = Select[oldStSt[[1]], #[[2]] > 0.01 &][[ ;; , 1]];
startingpoint = Table[#[[1]] → #[[2]] & /@ oldStSt[[i]], {i, 1, 2}];
paramsToScan = model[[4]];
paramNames = paramsToScan[[ ;; , 1]];
paramVals = paramsToScan[[ ;; , 2]];
paramLogExtremes = Table[
   var = 0.10;
   paramNames[[i]] → {(1 - var) * paramVals[[i]], (1 + var) * paramVals[[i]]},
   {i, 1, Length[paramNames]}];

paramLogVariance =
  #[[1]] → If[#[[2]] ≠ {0., 0.}, Variance[Log[RandomVariate[UniformDistribution[
          {#[[2, 1]], #[[2, 2]]}], 1000000000]]], 0] & /@ paramLogExtremes;

respTabA = Flatten[Table[
    param = model[[4, j]];
    tempMod = addParametersToModel[model, {param[[1]] → param[[2]] * 1.0001}];
    newStStUp = Quiet[ststSolMatFormR[tempMod, 10000, startingpoint]];
    respTab = Table[{param[[1]], vars[[i]], (param[[1]] /. paramLogVariance) *
        (((vars[[i]] /. newStStUp[[1]]) - (vars[[i]] /. oldStSt[[1]])) /
          (0.0001)) ^2}, {i, 1, Length[vars]}];
    respTab, {j, 1, Length[model[[4]]]}], 1];
```

In[∘]:=

```
overallVariances =
  Thread[vars → (Total[#] & /@ Table[Select[respTabA, #[[2]] == vars[[i]] &&
          ! #[[3]] === Indeterminate &], {i, 1, Length[vars]}][[ ;; ,  ;; , 3]])];

contributionOfPars = Table[
   num = Flatten[
      Select[respTabA, #[[1]] == paramNames[[i]] && #[[2]] == vars[[j]] &]][[3]];
   denom = vars[[j]] /. overallVariances;
   {paramNames[[i]], vars[[j]], num / denom * 100},
   {j, 1, Length[vars]}, {i, 1, Length[paramNames]}];
TableForm[contributionOfPars]
```

Out[∘]//TableForm=

model`A0<br>model`A[t]<br>0.	model`bM<br>model`A[t]<br>2.92516	model`betaX<br>model`A[t]<br>3.45507	model`c0<br>model`A[t]<br>0.00225865	model`c1<br>model`A[t]<br>0.0119772	model`eta<br>model`A[t<br>0.057024
model`A0<br>model`G[t]<br>0.	model`bM<br>model`G[t]<br>14.0638	model`betaX<br>model`G[t]<br>1.22941	model`c0<br>model`G[t]<br>0.000803703	model`c1<br>model`G[t]<br>0.0474471	model`eta<br>model`G[t<br>0.274192
model`A0<br>model`M[t]<br>0.	model`bM<br>model`M[t]<br>6.28617	model`betaX<br>model`M[t]<br>1.75225	model`c0<br>model`M[t]<br>0.00114551	model`c1<br>model`M[t]<br>0.0801215	model`eta<br>model`M[t<br>0.122549
model`A0<br>model`P[t]<br>0.	model`bM<br>model`P[t]<br>40.7835	model`betaX<br>model`P[t]<br>0.182329	model`c0<br>model`P[t]<br>0.000119192	model`c1<br>model`P[t]<br>0.000895461	model`eta<br>model`P[t<br>0.795023
model`A0<br>model`X[t]<br>0.	model`bM<br>model`X[t]<br>5.29066	model`betaX<br>model`X[t]<br>27.2662	model`c0<br>model`X[t]<br>0.0178244	model`c1<br>model`X[t]<br>0.0683581	model`eta<br>model`X[t<br>0.103134
model`A0<br>model`Y[t]<br>0.	model`bM<br>model`Y[t]<br>4.18512	model`betaX<br>model`Y[t]<br>5.64982	model`c0<br>model`Y[t]<br>0.0036934	model`c1<br>model`Y[t]<br>0.0137979	model`eta<br>model`Y[t<br>0.0815858

In[∘]:=

```
NotebookSave[];
```
